# Naringenin Regulates FKBP4/NR3C1/NRF2 Axis in Autophagy and Proliferation of Breast Cancer and Differentiation and Maturation of Dendritic Cell

**DOI:** 10.3389/fimmu.2021.745111

**Published:** 2022-01-11

**Authors:** Hanchu Xiong, Zihan Chen, Baihua Lin, Bojian Xie, Xiaozhen Liu, Cong Chen, Zhaoqing Li, Yunlu Jia, Zhuazhua Wu, Min Yang, Yongshi Jia, Linbo Wang, Jichun Zhou, Xuli Meng

**Affiliations:** ^1^ Cancer Center, Department of Radiation Oncology, Zhejiang Provincial People’s Hospital, Affiliated People’s Hospital, Hangzhou Medical College, Hangzhou, China; ^2^ Surgical Intensive Care Unit, First Affiliated Hospital, Zhejiang University, Hangzhou, China; ^3^ Department of Breast and Thyroid Surgery, Taizhou Hospital of Zhejiang Province, Taizhou, China; ^4^ Cancer Center, Department of Breast Surgery, Zhejiang Provincial People’s Hospital, People’s Hospital of Hangzhou Medical College, Hangzhou, China; ^5^ Department of Surgical Oncology, Sir Run Run Shaw Hospital, Zhejiang University, Hangzhou, China; ^6^ Department of Medical Oncology, First Affiliated Hospital, Zhejiang University, Hangzhou, China

**Keywords:** FKBP4, NRF2, NR3C1, autophagy, Dendritic cell, Breast cancer

## Abstract

NRF2 is an important regulatory transcription factor involved in tumor immunity and tumorigenesis. In this study, we firstly identified that FKBP4/NR3C1 axis was a novel negative regulator of NRF2 in human breast cancer (BC) cells. The effect of FKBP4 appeared to be at protein level of NRF2 since it could not suppress the expression of NRF2 at mRNA level. Bioinformatics analysis and *in vitro* experiments further demonstrated that FKBP4 regulated NRF2 *via* regulating nuclear translocation of NR3C1. We then reported that naringenin, a flavonoid, widely distributed in citrus and tomato, could suppress autophagy and proliferation of BC cells through FKBP4/NR3C1/NRF2 signaling pathway *in vitro* and *in vivo*. Naringenin was also found to promote dendritic cell (DC) differentiation and maturation through FKBP4/NR3C1/NRF2 axis. Therefore, our study found that naringenin could induce inhibition of autophagy and cell proliferation in BC cells and enhance DC differentiation and maturation, at least in part, though regulation of FKBP4/NR3C1/NRF2 signaling pathway. Identification of FKBP4/NR3C1/NRF2 axis would provide insights for novel anti-tumor strategy against BC among tumor microenvironment.

## Introduction

Breast cancer (BC) is a leading cause of cancer-related deaths in women aged 40 years and younger (1). Early detection and comprehensive treatments, which consist of surgery, radiation, chemotherapy, endocrine therapy and targeted therapy, have dramatically improved the prognosis of BC patients. In recent years, immunotherapy in BC showed promising future. Cancer vaccines, bispecific antibodies, and immune checkpoint inhibitors are verified to have potential applied value in BC immunotherapy (2). For instance, adaptive immune checkpoint therapies by targeting cytotoxic T-lymphocyte antigen-4 (CTLA-4), proNR3C1ammed cell death-1 (PD-1), and ligand partner for PD-1 (PD-L1) for BC have been used in clinical trial (3, 4). Nevertheless, a portion of BC patients still cannot benefit from the above-mentioned immunotherapy strategies (5). Therefore, unraveling the potential molecular mechanisms of the immune system in BC cells is essential to further understand and improve immune related anti-tumor effects.

The transcription factor nuclear factor erythroid 2-related factor 2 (NRF2), also known as NFE2L2, is a Cap’n’collar, leucine zipper transcription factor comprised of seven Neh domains, and is regarded as a significant orchestrator of the cellular antioxidant response (6). Recently, NRF2 has been found to be involved in innate immunity and cytokine secretion orchestrated by dendritic cell (DC) (7). Additionally, several studies revealed the suppressive function of NRF2 in tumorigenesis, namely, BC, gastric cancer, leukemia, prostate cancer, colorectal cancer, melanomas and so forth (8–13). The previous study of our team has summarized the biological crosstalk between innate Immunity and BC (14), and we are wondering whether and how NRF2 performs its anti-tumor and pro-immunity effects by connection with novel molecular chaperones in BC.

As one of the most extensively studied proteins among the 18 identified human FK506-binding proteins (FKBPs), FK506-binding protein 4 (FKBP4), also known as FKBP52, has been reported to exhibit multiple functions which involve binding to different cellular receptors or targets in various kinds of cancers, such as lung cancer (15), prostate cancer (16), and glioblastoma (17). For example, FKBP4 has been demonstrated to interact with heat shock protein 90 (Hsp90) to affect steroid hormone receptor function in BC (18). In terms of immune regulation, phytanoyl-CoA alpha-hydroxylase (PAHX) has been regarded as a specific target of FKBP4 for studying the cellular signaling pathway in the presence of immunosuppressant drugs (19). Our previous work found that FKBP4 interacted with non-coding RNAs and mRNAs during the occurrence and development of BC, thus playing a role in promoting cancer (20, 21). Nevertheless, current immunologic mechanism of FKBP4 is still in its infancy for BC, and it is necessary to explore more new detailed contents of its regulation of innate and adaptive immunity functions during the occurrence and development of BC.

The NR3C1 (nuclear receptor subfamily 3, group C, member 1/glucocorticoid receptor) normally resides in the cell cytoplasm, where the NR3C1 protein translocates to the nucleus when bound to glucocorticoids, and thus is involved in growth, reproduction, metabolism, immune and inflammatory reactions, and cardiovascular functions and tumor cellular proliferation and differentiation (22). Researches on NR3C1 and BC have also been conducted in recent years, e.g., high levels of NR3C1 expression and high concentrations of cortisol have been shown to have an anti-proliferative effect in cancerous breast tissue (23). Some studies have preliminarily found that NR3C1 is associated with FKBPs, but the specific mechanisms remain unclear in BC (24).

In this study, we showed that naringenin, a flavonoid shown to have anti-tumor effects in various carcinomas in other studies (25), suppressed both autophagy and proliferation *via* new-found FKBP4/NR3C1/NRF2 signaling pathway in luminal A and basal-like subtype of BC cells. Mechanically, these effects relied on downregulation of FKBP4, which post-transcriptionally upregulated NRF2 through intensive nuclear translocation of NR3C1. Additionally, naringenin could promote differentiation and maturation of DC among tumor microenvironment through regulating FKBP4/NR3C1/NRF2 signaling pathway. Identification of FKBP4/NR3C1 axis as the novel NRF2 regulator would provide in-depth insights for immunological anti-tumor strategy to overcome BC.

## Materials and Methods

### Cell Culture

MCF10A, MCF7, T47D, BT549, MDA231, and SKBR3 cells were obtained from the American Type Culture Collection (ATCC). MCF10A was cultured in Mammary Epithelial Basal medium. MCF7 was cultured in Eagle’s Minimum Essential medium. T47D and SKBR3 were cultured in Dulbecco’s modified Eagle’s medium. BT549 was cultured in Roswell Park Memorial Institute (RPMI) medium. MDA231 was cultured in Leibovitz’s L15 medium. Growth media were supplemented with 10% fetal calf serum and penicillin/streptomycin (100 units per ml). All human cell lines were cultured at 37°C in a humidified incubator supplied with 5% CO_2_.

### Antibodies and Reagents

Antibodies were used in the following dilutions: NRF2 (1:1,000, Proteintech, #16396-1-AP), FKBP4 (1:1,000, Proteintech, #10655-1-AP), NR3C1 (1:1,000, Proteintech, #24050-1-AP), P62 (1:1,000, MBL, #PM045), Histone (1:1,000, CST, #3638), GAPDH (1:1,000, Proteintech, #60004-1-Ig), Flag (1:1,000, Sigma, #F3165), HA (1:1,000, Biolegend, #901514), Secondary antibody goat anti-mouse (1:2,500, HuaBio, #HA1006), secondary antibody goat anti-rabbit (1:2,500, HuaBio, #HA1001), Anti-RABBIT IgG (H&L) (GOAT) Antibody Rhodamine Conjugated (1:200, MULTISCIENCES, #RK-611-1002), Anti-RABBIT IgG (H&L) (GOAT) Antibody ATTO 488 Conjugated (1:200, MULTISCIENCES, #RK-611-152-122S), Anti-Mouse CD11c, PE (1:20, MULTISCIENCES, #AM011C04), Anti-Mouse CD86 (B7-2), APC (AM08605). Naringenin was purchased from APExBIO (#N1370), and resolved in DMSO at 10 mM. BC cells were treated by medium with naringenin for different points in time.

### Gene Silence

The shRNA against NRF2 was purchased from RIBBIO (Guangzhou, China), and shRNA against NR3C1 was purchased from Shanghai Generay Biotech Co., Ltd. The shRNA targeting sequences of NRF2 (26) and NR3C1 (27) were from published articles, lentiviral particles were produced as follows. In brief, HEK293T packaging cells were transfected with 800 ng pLKO.1 DNA in combination with the packaging plasmids 200 ng lenti-VSV-G, 400 ng lenti-RRE, and 140 ng lenti-REV. Virus containing supernatant was harvested at 36 and 48 h after transfection and filtered through a 0.45 μM syringe filter with the addition of 10 μM DEAE. Supernatants were used to infect target cells in another 12 h period.

### Western Blotting and co-IP

Knockdown and overexpression efficiencies and biochemical responses were analyzed by western blotting. Cells were lysed in RIPA lysis buffer (EMD Millipore Corp.), supplemented with protease inhibitor and phosphatase inhibitor cocktail tablets (Roche). Separated proteins were transferred to nitrocellulose filter membranes and blocked in 5% milk in Tris-buffered saline, with 0.05% Tween-20. Immunodetection was done with various primary antibodies. Appropriate horseradish peroxidase-conjugated secondary antibodies were used and signals were visualized with Bio-Rad chemiluminescence by Bio-Rad ChemiDoc™ MP Imaging System. Cells for co-IP were lysed in lysis buffer (50 mM Tris, pH 7.5, with 150 mM NaCl, 0.5% NP-40, and protease inhibitor and phosphatase inhibitor cocktail tablets (Roche) at 4°C for 30 min. After sonication and centrifugation, cell lysates were incubated with beads (Sigma) at 4°C overnight on a rotator. After six washes with wash buffer (20 mM Tris, pH 7.5, 100 mM NaCl, 0.05% Tween-20, 0.1 mM EDTA), 50 μl of elution buffer (100 mM glycine-HCl, pH 2.5) was added to resuspend the beads, and the eluted proteins were obtained by centrifugation, followed by SDS-PAGE and immunoblotting analysis.

### Quantitative RT-qPCR

Total RNA was extracted from cells by using TRIzol (Invitrogen). Reverse transcription was carried out with a 40-μl volume by using a PrimeScript™ RT Master Mix kit (TaKaRa) according to the manufacturer’s instructions. Quantitative real-time PCR (qPCR) was carried out on an Applied Biosystems Fast 7500 machine by using a TB NR3C1een^®^ Premix Ex Taq™ II kit (TaKaRa) and the following primer sets were used for qPCR analysis: NRF2, 5′-GAGAGCCCAGTCTTCATTGC-3′ (forward) and 5′-TTGGCTTCTGGACTTGGAAC-3′ (reverse), FKBP4, 5′- CATTGCCATAGCCACCATGAA-3′ (forward) and 5′-TCCAGTGCAACCTCCACGATA-3′ (reverse), NR3C1, 5′-AGTGGTTGAAAATCTCCTTAACTATTGCT-3′ (forward) and 5′-GGTATCTGATTGGTGATGATTTCAGCTA-3′ (reverse), GAPDH, 5′-ATGACATCAAGAAGGTGGTG-3′ (forward) and 5′-CATACCAGGAAATGAGCTTG-3′ (reverse) as a control. The qPCR assay was carried out with a 15-μl volume consisting of 7.5 μl of a 2× TB NR3C1een mix solution, 0.3 μl of 10 μM of each oligonucleotide primer, 0.3 μl of ROX Reference Dye II and 2 μl of the cDNA template. Target fragment amplification was carried out as follows: 95°C for 30 s, followed by 40 cycles consisting of 95°C for 5 s and 60°C for 34 s. Melting-curve analysis was carried out at 90°C for 15 s and then at 60°C for 1 min and 95°C for 15 s.

### Plasmids

FKBP4-HA, NR3C1-HA, NRF2-Flag, and the control plasmid were constructed by Shanghai Generay Biotech Co., Ltd.

### Immunofluorescence

T47D or BT549 cells grown on coverslips were fixed for 15 min with 4% paraformaldehyde in PBS, permeabilized for 10 min in 0.1% Triton X-100 in PBS, and blocked using 5% BSA for 1 h. The cells were then incubated with primary antibodies at 4°C overnight. After a rinse with PBS, the cells were incubated with fluorescent-conjugated secondary antibodies for 1 h at 37°C. The nuclei were counterstained with 4, 6-diamidino-2-phenylindole (DAPI; Sigma–Aldrich). Images were captured using a ZEISS laser scanning confocal microscope (LSM710; Zeiss). ZEISS ZEN Microscope software was used for acquisition.

### Autophagy Flux Monitoring

To evaluate the formation of fluorescent LC3B puncta, p−mCherry−C1−EGFP−hLC3B (LC3B) was used to monitor autophagy flux, 48 h after LC3B co−transfection with siRNAs, the cells were washed with 1× PBS and immediately analyzed *via* confocal microscopy (magnification, 500×). The nuclei were counterstained with 4, 6-diamidino-2-phenylindole (DAPI; Sigma-Aldrich). Images were captured using a ZEISS laser scanning confocal microscope (LSM710; Zeiss). ZEISS ZEN Microscope software was used for acquisition.

### Nuclear and Cytoplasmic Fractionation

T47D or BT549 cells were transfected with the indicated siRNAs for 72 h, then cells were harvested, and the nuclear and cytoplasmic fractions were separated using Thermo Fisher Scientific NE-PER Nuclear and Cytoplasmic Extraction Reagents (78833) according to the manufacturer’s protocol.

### Transient Transfection

Breast cancer cells cultured in 12-well tissue culture plates were transiently transfected with plasmids using Lipofectamine^®^ 2000 Reagent (Invitrogen) or siRNAs using Lipofectamine^®^ RNAiMAX Reagent (Invitrogen) as instructed by the manufacturer. The siRNA targeting FKBP4, NR3C1, and NRF2, and also negative control siRNA were purchased from RIBBIO (Guangzhou, China). Seventy two hours later, the whole-cell extract was prepared for RT-qPCR or western blot analysis.

### Cell Proliferation Assay

Cell proliferation was analyzed using a Cell Counting Kit-8 (CCK-8) (DOJINDO). All cells were seeded into 96-well plates at a density of 5,000 cells/well in a 100 µl volume and incubated at 37°C under 5% CO_2_ for 24, 48, and 72 h, followed by the addition of 10 μl of CCK-8 solution. The absorbance in each well was measured after 1 h incubation using a microculture plate reader at a test wavelength of 450 nm. Three replicate wells were set up in each group, and three independent experiments were performed.

### Colony Formation Assay

Five hundred cells per well of breast cancer were seeded in a 6-well plate for colony formation assay. Two weeks after, they were fixed with 4% paraformaldehyde and stained with Crystal Violet. Colonies were quantified using ImageJ software.

### Generation of Bone Marrow Cells

The DC cells were purchased from Wuhan Procell Life Science&Technology Co., Ltd. The cells were cultured in RPMI medium with 10% FBS, penicillin/streptomycin (100 units per ml), GMCSF (20 ng/ml) (Signalway Antibody, #AP73338), and IL4 (10 ng/ml) (Signalway Antibody, #AP73338).

### Flow Cytometry Analysis

Single cell suspensions were surface-labeled using the mAbs mentioned above for 30 min at 4°C. After using Flow cytometry Staining buffer (MULTI SCIENCES, China), cells were analyzed with a BD FACSCalibur Flow Cytometer (Becton Dickinson). Cytometry data was analyzed using FlowJo software version 10 (CD11c gating was firstly used to screen for DC cells, and then CD86 gating was finally focused).

### *In Vivo* Tumor Xenograft Assays

Six-week-old male BALB/c nude mice were purchased from Vital River Laboratory Animal Technology Co, Ltd (Beijing, P.R. China). Animal experimental procedures were approved by the Medical Ethics Committee of Zhejiang Provincial People’s Hospital. The six-week-old male mice were randomized into different groups. T47D shControl and T47D shNRF2 cells (5 × 10^6^ cells/mice) were implanted subcutaneously into the flank of nude mice. After tumor formation, naringenin (50 mg/kg) was administered orally to its respective animal treatment groups for 28 days. Tumor volume (mm^3^) was measured every three days and calculated by the formula (length × width × width)/2. When the tumors had reached a volume of approximately 600 mm^3^, the mice were euthanized.

### Bioinformatics Analysis

The localizations of FKBP4, NR3C1, and NRF2 protein were generated by PROTTER database (28) (https://wlab.ethz.ch/protter/start/). The immunofluorescence stainings of the subcellular distribution of FKBP4, NR3C1, and NRF2 protein were generated by from the HPA database (https://www.proteinatlas.org/). The expression, correlation, and prognostic module of the Breast Cancer Gene-Expression Miner v4.7 database (bc-GenExMiner v4.7) (29) (bcgenex.centregauducheau.fr) were used to evaluate the expression, correlation and prognostic merit of FKBP4, NR3C1, and NRF2 in human breast cancer. The lymphocyte, immune subtype, and molecular subtype module of the TISIDB database (30) (http://cis.hku.hk/TISIDB/), the gene, survival and scna module of the TIMER database (31) (https://cistrome.shinyapps.io/timer/) were used to evaluate relation between DC and immunological merit of FKBP4, NR3C1, and NRF2 in human breast cancer. Transcription factors in NRF2 promoter were predicted by PROMO (http://alggen.lsi.upc.es/cgi-bin/promo_v3/promo/promoinit.cgi?dirDB=TF_8.3). Protein to protein interacting network was analyzed by STRING (https://string-db.org/).

### Statistics

Two-tailed Student’s t-test was used in this study. Data shown was mean ± SD from at least three independent experiments. Statistical probability was expressed as *p <0.05, **p <0.01, and ***p <0.001.

## Results

### Negative Correlation of FKBP4 and NRF2 in Breast Cancer

**Figure 1:**
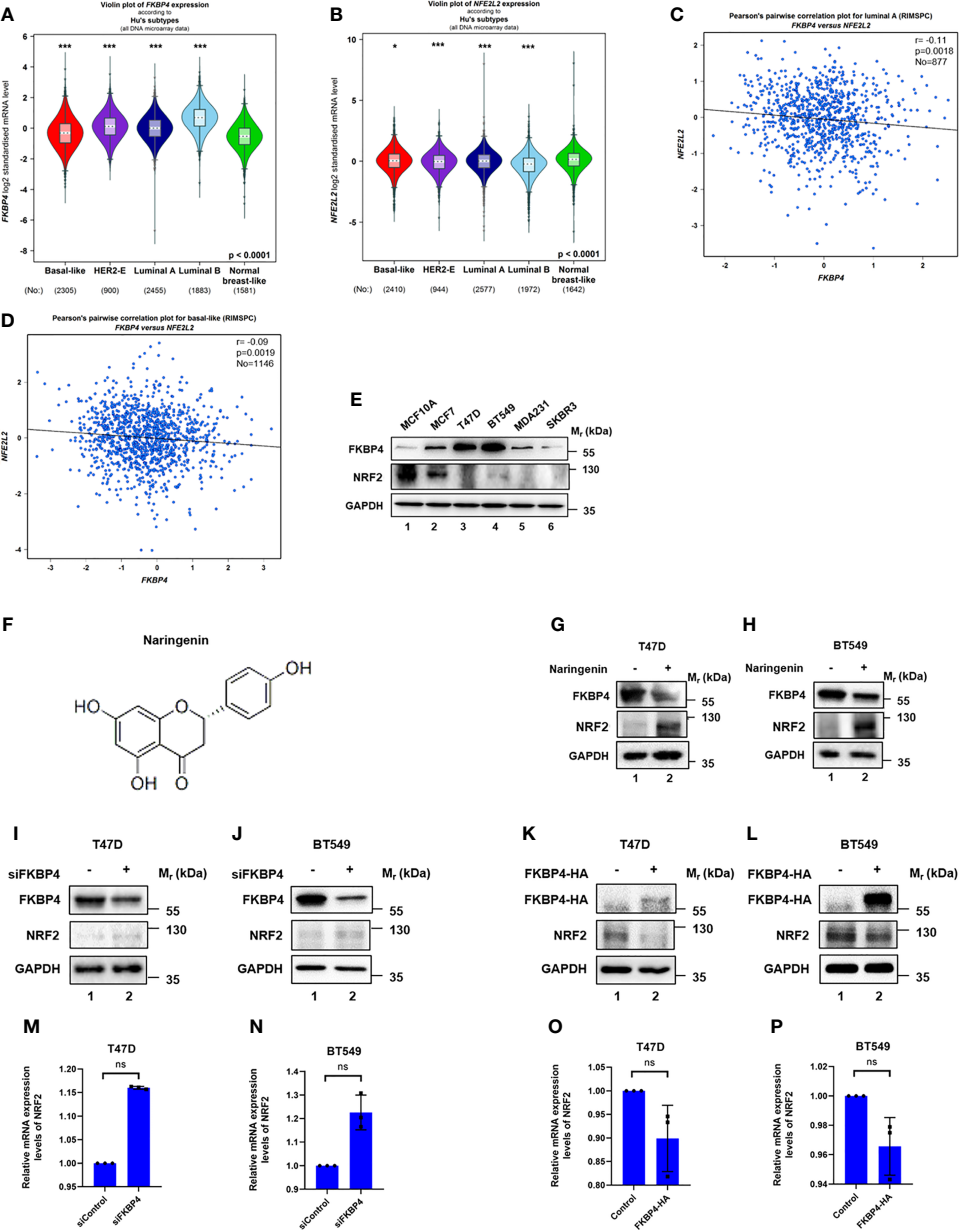
FKBP4 negatively regulates NRF2 at protein level. **(A)** A violin plot indicated upregulated FKBP4 in luminal A, luminal B, HER2-positive and basal-like subtype of BC patients than the normal group. **(B)** A violin plot indicated downregulated NRF2 in luminal A, luminal B, HER2-positive and basal-like subtype of BC patients than the normal group. **(C)** Pearson’s pairwise correlation plot of FKBP4 and NRF2 in luminal A subtype of BC patients, r = −0.11, p = 0.0018, No = 877. **(D)** Pearson’s pairwise correlation plot of FKBP4 and NRF2 in basal-like subtype of BC patients, r = −0.09, p = 0.0019, No = 1146. **(E)** Representative western blot analysis results of FKBP4, NRF2, and endogenous control GAPDH. Western blot analysis showed a negatively correlated expression of FKBP4 and NRF2 in BC and normal breast cells. **(F)** Chemical structure of naringenin. **(G, H)** Representative western blot analysis results of FKBP4, NRF2, and endogenous control GAPDH. Western blot analysis showed decreased FKBP4 expression and increased NRF2 expression in T47D and BT549 cells treated by 100 nM naringenin for 48 h **(I, J)** Representative western blot analysis results of FKBP4, NRF2, and endogenous control GAPDH. Western blot analysis showed silencing FKBP4 resulted in upregulation of NRF2 in T47D and BT549 cells. **(K, L)** Representative western blot analysis results of FKBP4-HA, NRF2, and endogenous control GAPDH. Western blot analysis showed overexpressing FKBP4 resulted in downregulation of NRF2 in T47D and BT549 cells. **(M, N)** RT-qPCR showed silencing FKBP4 resulted in no significant changes of NRF2 in T47D and BT549 cells (n = 3 independent biological replicates). **(O, P)** RT-qPCR showed overexpressing FKBP4 resulted in no significant changes of NRF2 in T47D and BT549 cells (n = 3 independent biological replicates). *p <0.05, ***p <0.001, ns, nonsense.

Firstly, the FKBP4 and NRF2 protein topology both revealed intracellular membrane (cytosol and nucleoplasm) localization (**Figures S1A–D**), we also observed that FKBP4 and NRF2 colocalized with the nuclear marker in different cells by immunofluorescence assay of the HPA database, suggesting the subcellular localization of FKBP4 and NRF2 in nuclei (**Figures S1E, F**). Then we used bc-GenExMiner v4.7 database, an online public tool focused on BC, to explore the clinicopathological characteristics of FKBP4 and NRF2. Upregulated FKBP4 was found significantly related to luminal A, luminal B, HER2-positive and basal-like subtype of BC patients than the normal group (**Figure 1A**), and downregulated NRF2 was significantly related to luminal A, luminal B, HER2-positive and basal-like subtype patients than the normal group (**Figure 1B**), and the same results for TISIDB database (**Figure S2**). Furthermore, we validated a significant negative association between FKBP4 and NRF2 both in luminal A and basal-like subtype of BC patients (**Figures 1C, D**), but not in luminal B and HER2-positive subtype of BC patients (**Figure S3**). The prognostic merits of FKBP4 and NRF2 in luminal A and basal-like subtype of BC patients were further analyzed by using bc-GenExMiner v4.7, the Kaplan–Meier curve showed that increased levels of FKBP4 and decreased levels of NRF2 were strongly correlated with worse survival both in luminal A and basal-like subtype of BC patients (**Figure S4**).

We also tested five BC cell lines (MCF7, T47D, BT549, MDA231, and SKBR3) and also normal breast cell line (MCF10A) for expression levels of FKBP4 and NRF2. As shown in **Figure 1E**, expression of FKBP4 and NRF2 were negatively correlated in BC cell lines, especially in T47D (representing luminal A subtype of BC) and BT549 (representing basal-like subtype of BC) cell lines. Since naringenin (shown in **Figure 1F**) has been reportedly involved in immunity regulation (32), we wondered its effect of pharmacologically modulating NRF2 expression. As shown in **Figures 1G, H**, naringenin significantly decreased FKBP4 expression and increased NRF2 expression in T47D and BT549 cells.

### FKBP4 Negatively Regulates NRF2 at Protein Level

To confirm the specific interaction between FKBP4 and NRF2 at the molecular level, we used siRNA specifically targeting FKBP4 in T47D and BT549 cells, which led to upregulation of NRF2 at protein level (**Figures 1I, J**). In addition, we transfected FKBP4-HA plasmid in T47D and BT549 cells, which resulted in downregulation of endogenous NRF2 (**Figures 1K, L**). These results clearly indicated that FKBP4 had a role in negatively regulating NRF2 protein expression. However, we found no evidence that silencing or overexpressing FKBP4 could lead to changes of NRF2 at mRNA level in T47D and BT549 cells (**Figures 1M–P**).

### Negative Correlation of FKBP4 and NR3C1 in Breast Cancer

**Figure 2:**
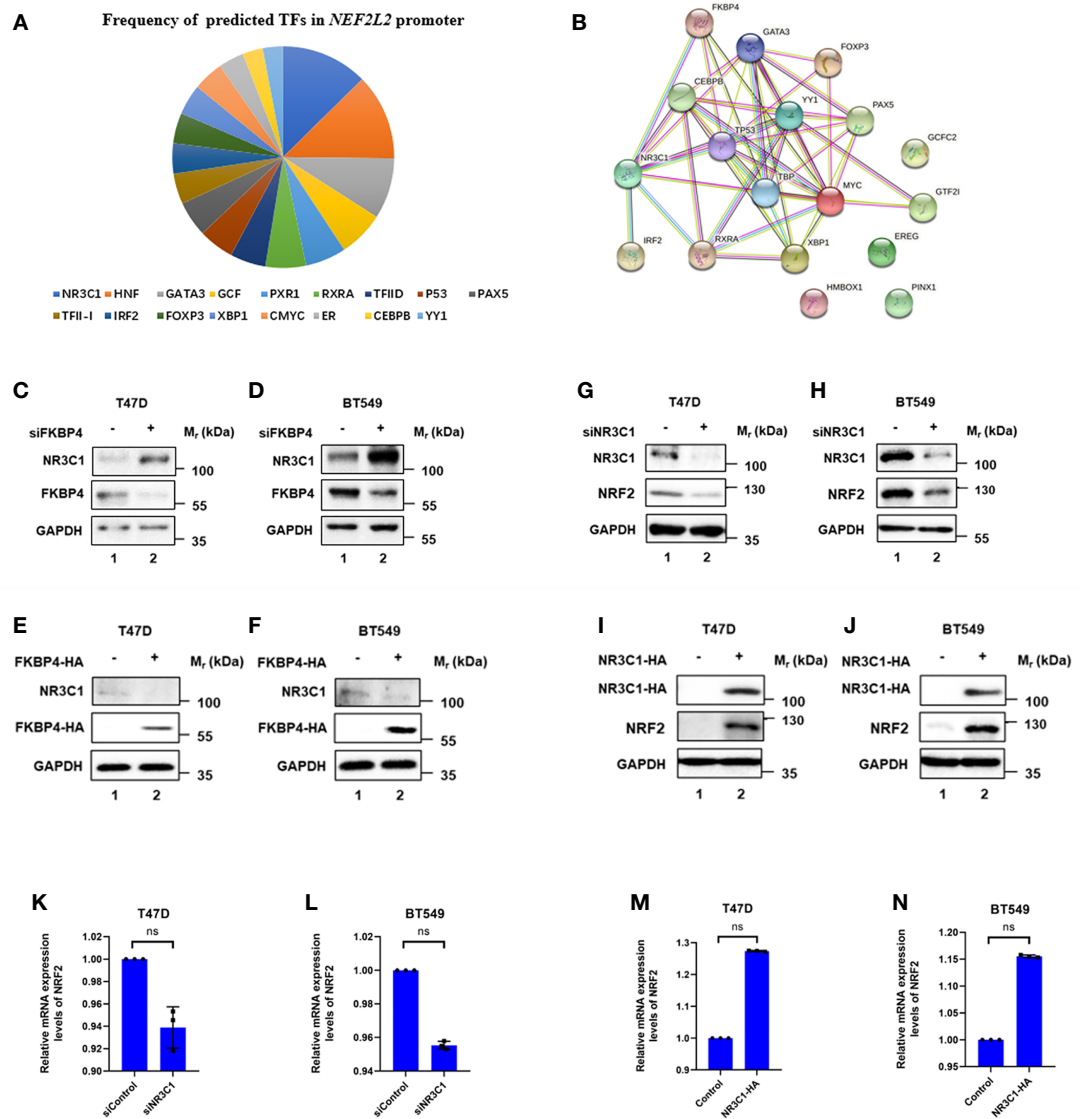
FKBP4 regulates NRF2 *via* NR3C1. **(A)** Pie chart showed frequency of 17 predicted transcription factors in NRF2 promoter. **(B)** Protein to protein interacting network of FKBP4 and 17 putative NRF2 transcription factors. **(C–F)** Representative western blot analysis results of FKBP4, NR3C1, and endogenous control GAPDH. Western blot analysis showed silencing FKBP4 resulted in upregulation of NR3C1, overexpressing FKBP4 resulted in downregulation of NR3C1 in T47D and BT549 cells. **(G–J)** Representative western blot analysis results of NR3C1, NRF2, and endogenous control GAPDH. Western blot analysis showed silencing NR3C1 resulted in downregulation of NRF2, overexpressing NR3C1 resulted in upregulation of NRF2 in T47D and BT549 cells. **(K–N)** RT-qPCR showed silencing NR3C1 resulted in no significant changes of NRF2, overexpressing NR3C1 resulted in no significant changes of NRF2 in T47D and BT549 cells (n = 3 independent biological replicates). ns, nonsense.

We then continued to figure out potential factors involved in the FKBP4/NRF2 axis. Firstly, we used PROMO (33, 34), a virtual laboratory for the identification of putative transcription factors (TFs) binding sites in DNA sequences, to find predicted TFs binding to NRF2. After inputting NRF2 promoter sequence, namely, 1,000 bases upstream, CDS exons, and 150 bases downstream in PROMO (**Table 1**), 17 TFs were shown in order of frequency: NR3C1, HNF, GATA3, GCF, PXR1, RXRA, TFIID, P53, PAX5, TFII-I, IRF2, FOXP3, XBP1, cMyc, ER, CEBPB, and YY1 (**Figure 2A**). Besides FKBP4 was shown to connect to NR3C1 with a highest score in protein to protein interacting network on STRING database (35) (**Figure 2B**) (**Table 2**), we wondered whether NR3C1 was involved in FKBP4 associated NRF2 dysregulation. The NR3C1 protein topology also revealed intracellular membrane (cytosol and nucleoplasm) localization (**Figures S5A, B**), immunofluorescence assay of the HPA database suggested the subcellular localization of NR3C1 in nuclei (**Figure S5C**).

**Table 1:** NRF2 promoter sequence including 1,000 bases upstream, CDS exons, and 150 bases downstream.

AAGGGCCCGGACTCTTGCCCCGCCCTTGTGGGGCGGGAGGCGGAGCGGGGCAGGGGCCCGCCGGCGTGTAGCCGATTACCGAGTGCCGGGGAGCCCGGAGGAGCCGCCGACGCAGCCGCCACCGCCGCCGCCGCCGCCACCAGAGCCGCCCTGTCCGCGCCGCGCCTCGGCAGCCGGAACAGGGCCGCCGTCGGGGAGCCCCAACACACGGTCCACAGCTCATCATGATGGACTTGGAGCTGCCGCCGCCGGGACTCCCGTCCCAGCAGGTGCTGCCTCGGCCCTCTGGGCCCTGCGGTGGTGCCGGGACGGGGGCGGGGGAGCTAGGGAACCGCGGCCCGGGAGGGACAGCCGCAACCGGCTTCCCCCACGTCTGGCCAGAGCCAGGACCGCGGCGCTGGGTAGAGCCGCCGCGCTTGCGCCGGGGCAGGGCGGGGAGGGGCAGCGGGGACGCGGCCGGGTGATCCGACCGACCACGAGCCCGAGGGCGAACGGGTGGGAAGTTGCGGGAAGGTCTGGGGACTGAGCCCGCTCGCGTGGGCCTTGGGGGAGAATCCAGCCGCGTCCCCGGGCCCGAGAGCTGGGACTCCGGAGCCCCTAAGTTTGAGCGGCCCGGTGGGCGGCGGGGCAAGAGGGGGCGGACGCTGGCCGTCTGAGCCGGCGCGGCCCGGCCCTTCCGGGGCTGCGCGGCTCCCCCGCCTCGGTGCCGGCAAAAATGTGCCTAGTCACGGGGCCGCTCTCGGGGGAACTGAGGTCGCCTTCGGGCTGGGACCCGGAGCCCCTTCGCCGCGCCCCAAGACCTCCTTGAGTGCGGGCTGCGACGCGCTCACCCCGCTGGGCCGTCTGTGGGCGCGGCTTTGCGAAGTCATCCATCTCTCGGATCACTCTCTGGCAGCCTTGAGCTCTCTTGAAAGCCCAGCCCCGGGACGAGGGAGGAGCGCCTTAAGTGCCCAGCGGGCTCAGAAGCCCCGACGTGTGGCGGCTGAGCCGGGCCCCGCGCATGGATTTGATTGACATACTTTGGAGGCAAGATATAGATCTTGGAGTAAGTCGAGAAGTATTTGACTTCAGTCAGCGACGGAAAGAGTATGAGCTGGAAAAACAGAAAAAACTTGAAAAGGAAAGACAAGAACAACTCCAAAAGGAGCAAGAGAAAGCCTTTTTCGCTCAGTTACAACTAGATGAAGAGACAGGTGAATTTCTCCCAATTCAGCCAGCCCAGCACATCCAGTCAGAAACCAGTGGATCTGCCAACTACTCCCAGGTTGCCCACATTCCCAAATCAGATGCTTTGTACTTTGATGACTGCATGCAGCTTTTGGCGCAGACATTCCCGTTTGTAGATGACAATGAGGTTTCTTCGGCTACGTTTCAGTCACTTGTTCCTGATATTCCCGGTCACATCGAGAGCCCAGTCTTCATTGCTACTAATCAGGCTCAGTCACCTGAAACTTCTGTTGCTCAGGTAGCCCCTGTTGATTTAGACGGTATGCAACAGGACATTGAGCAAGTTTGGGAGGAGCTATTATCCATTCCTGAGTTACAGTGTCTTAATATTGAAAATGACAAGCTGGTTGAGACTACCATGGTTCCAAGTCCAGAAGCCAAACTGACAGAAGTTGACAATTATCATTTTTACTCATCTATACCCTCAATGGAAAAAGAAGTAGGTAACTGTAGTCCACATTTTCTTAATGCTTTTGAGGATTCCTTCAGCAGCATCCTCTCCACAGAAGACCCCAACCAGTTGACAGTGAACTCATTAAATTCAGATGCCACAGTCAACACAGATTTTGGTGATGAATTTTATTCTGCTTTCATAGCTGAGCCCAGTATCAGCAACAGCATGCCCTCACCTGCTACTTTAAGCCATTCACTCTCTGAACTTCTAAATGGGCCCATTGATGTTTCTGATCTATCACTTTGCAAAGCTTTCAACCAAAACCACCCTGAAAGCACAGCAGAATTCAATGATTCTGACTCCGGCATTTCACTAAACACAAGTCCCAGTGTGGCATCACCAGAACACTCAGTGGAATCTTCCAGCTATGGAGACACACTACTTGGCCTCAGTGATTCTGAAGTGGAAGAGCTAGATAGTGCCCCTGGAAGTGTCAAACAGAATGGTCCTAAAACACCAGTACATTCTTCTGGGGATATGGTACAACCCTTGTCACCATCTCAGGGGCAGAGCACTCACGTGCATGATGCCCAATGTGAGAACACACCAGAGAAAGAATTGCCTGTAAGTCCTGGTCATCGGAAAACCCCATTCACAAAAGACAAACATTCAAGCCGCTTGGAGGCTCATCTCACAAGAGATGAACTTAGGGCAAAAGCTCTCCATATCCCATTCCCTGTAGAAAAAATCATTAACCTCCCTGTTGTTGACTTCAACGAAATGATGTCCAAAGAGCAGTTCAATGAAGCTCAACTTGCATTAATTCGGGATATACGTAGGAGGGGTAAGAATAAAGTGGCTGCTCAGAATTGCAGAAAAAGAAAACTGGAAAATATAGTAGAACTAGAGCAAGATTTAGATCATTTGAAAGATGAAAAAGAAAAATTGCTCAAAGAAAAAGGAGAAAATGACAAAAGCCTTCACCTACTGAAAAAACAACTCAGCACCTTATATCTCGAAGTTTTCAGCATGCTACGTGATGAAGATGGAAAACCTTATTCTCCTAGTGAATACTCCCTGCAGCAAACAAGAGATGGCAATGTTTTCCTTGTTCCCAAAAGTAAGAAGCCAGATGTTAAGAAAAACTAGTTTGTTTGGGATTCCTTTGAGTTTATTCACACAGTATTTTCCATAAGATAATCATCCATTTAATTTAGATGTTTTGCATTTTCAGAATGTTTTATTTGAAAATATAAAAATAATTTTATACTACATTTCTAGTACCAGGGTTACAGAGAA

**Table 2:** Protein to protein interacting score of FKBP4 and predicted NRF2 transcription factors.

node1	node2	node1 accession	node2 accession	score
TP53	TBP	ENSP00000269305	ENSP00000375942	0.999
TBP	TP53	ENSP00000375942	ENSP00000269305	0.999
NR3C1	FKBP4	ENSP00000231509	ENSP00000001008	0.999
FKBP4	NR3C1	ENSP00000001008	ENSP00000231509	0.999
YY1	TP53	ENSP00000262238	ENSP00000269305	0.994
TP53	YY1	ENSP00000269305	ENSP00000262238	0.994
YY1	MYC	ENSP00000262238	ENSP00000479618	0.983
MYC	YY1	ENSP00000479618	ENSP00000262238	0.983
TP53	NR3C1	ENSP00000269305	ENSP00000231509	0.979
NR3C1	TP53	ENSP00000231509	ENSP00000269305	0.979
MYC	CEBPB	ENSP00000479618	ENSP00000305422	0.97
GATA3	FOXP3	ENSP00000368632	ENSP00000365380	0.97
FOXP3	GATA3	ENSP00000365380	ENSP00000368632	0.97
CEBPB	MYC	ENSP00000305422	ENSP00000479618	0.97
YY1	CEBPB	ENSP00000262238	ENSP00000305422	0.95
CEBPB	YY1	ENSP00000305422	ENSP00000262238	0.95
RXRA	MYC	ENSP00000419692	ENSP00000479618	0.931
MYC	RXRA	ENSP00000479618	ENSP00000419692	0.931
TP53	MYC	ENSP00000269305	ENSP00000479618	0.923
RXRA	NR3C1	ENSP00000419692	ENSP00000231509	0.923
NR3C1	RXRA	ENSP00000231509	ENSP00000419692	0.923
MYC	TP53	ENSP00000479618	ENSP00000269305	0.923
TBP	MYC	ENSP00000375942	ENSP00000479618	0.918
MYC	TBP	ENSP00000479618	ENSP00000375942	0.918
NR3C1	IRF2	ENSP00000231509	ENSP00000377218	0.914
IRF2	NR3C1	ENSP00000377218	ENSP00000231509	0.914
MYC	GTF2I	ENSP00000479618	ENSP00000460070	0.902
GTF2I	MYC	ENSP00000460070	ENSP00000479618	0.902
TBP	GTF2I	ENSP00000375942	ENSP00000460070	0.884
GTF2I	TBP	ENSP00000460070	ENSP00000375942	0.884
NR3C1	CEBPB	ENSP00000231509	ENSP00000305422	0.879
CEBPB	NR3C1	ENSP00000305422	ENSP00000231509	0.879
XBP1	TBP	ENSP00000216037	ENSP00000375942	0.846
TBP	XBP1	ENSP00000375942	ENSP00000216037	0.846
YY1	GTF2I	ENSP00000262238	ENSP00000460070	0.786
GTF2I	YY1	ENSP00000460070	ENSP00000262238	0.786
YY1	RXRA	ENSP00000262238	ENSP00000419692	0.752
RXRA	YY1	ENSP00000419692	ENSP00000262238	0.752
TBP	GATA3	ENSP00000375942	ENSP00000368632	0.732
GATA3	TBP	ENSP00000368632	ENSP00000375942	0.732
YY1	GATA3	ENSP00000262238	ENSP00000368632	0.685
GATA3	YY1	ENSP00000368632	ENSP00000262238	0.685
TP53	CEBPB	ENSP00000269305	ENSP00000305422	0.67
CEBPB	TP53	ENSP00000305422	ENSP00000269305	0.67
PAX5	MYC	ENSP00000350844	ENSP00000479618	0.664
MYC	PAX5	ENSP00000479618	ENSP00000350844	0.664
XBP1	PAX5	ENSP00000216037	ENSP00000350844	0.652
XBP1	CEBPB	ENSP00000216037	ENSP00000305422	0.652
PAX5	XBP1	ENSP00000350844	ENSP00000216037	0.652
CEBPB	XBP1	ENSP00000305422	ENSP00000216037	0.652
YY1	PAX5	ENSP00000262238	ENSP00000350844	0.65
PAX5	YY1	ENSP00000350844	ENSP00000262238	0.65
TP53	PAX5	ENSP00000269305	ENSP00000350844	0.645
PAX5	TP53	ENSP00000350844	ENSP00000269305	0.645
TP53	FKBP4	ENSP00000269305	ENSP00000001008	0.631
FKBP4	TP53	ENSP00000001008	ENSP00000269305	0.631
MYC	GATA3	ENSP00000479618	ENSP00000368632	0.626
GATA3	MYC	ENSP00000368632	ENSP00000479618	0.626
TP53	GATA3	ENSP00000269305	ENSP00000368632	0.6
GATA3	TP53	ENSP00000368632	ENSP00000269305	0.6
PAX5	GATA3	ENSP00000350844	ENSP00000368632	0.593
GATA3	PAX5	ENSP00000368632	ENSP00000350844	0.593
YY1	TBP	ENSP00000262238	ENSP00000375942	0.582
TBP	YY1	ENSP00000375942	ENSP00000262238	0.582
TP53	FOXP3	ENSP00000269305	ENSP00000365380	0.576
FOXP3	TP53	ENSP00000365380	ENSP00000269305	0.576
NR3C1	MYC	ENSP00000231509	ENSP00000479618	0.575
MYC	NR3C1	ENSP00000479618	ENSP00000231509	0.575
MYC	FOXP3	ENSP00000479618	ENSP00000365380	0.569
FOXP3	MYC	ENSP00000365380	ENSP00000479618	0.569
TBP	PAX5	ENSP00000375942	ENSP00000350844	0.562
PAX5	TBP	ENSP00000350844	ENSP00000375942	0.562
TBP	NR3C1	ENSP00000375942	ENSP00000231509	0.546
NR3C1	TBP	ENSP00000231509	ENSP00000375942	0.546
XBP1	TP53	ENSP00000216037	ENSP00000269305	0.526
TP53	XBP1	ENSP00000269305	ENSP00000216037	0.526
XBP1	MYC	ENSP00000216037	ENSP00000479618	0.515
MYC	XBP1	ENSP00000479618	ENSP00000216037	0.515
GATA3	CEBPB	ENSP00000368632	ENSP00000305422	0.508
CEBPB	GATA3	ENSP00000305422	ENSP00000368632	0.508
RXRA	CEBPB	ENSP00000419692	ENSP00000305422	0.487
CEBPB	RXRA	ENSP00000305422	ENSP00000419692	0.487
XBP1	GATA3	ENSP00000216037	ENSP00000368632	0.468
GATA3	XBP1	ENSP00000368632	ENSP00000216037	0.468
MYC	FKBP4	ENSP00000479618	ENSP00000001008	0.467
FKBP4	MYC	ENSP00000001008	ENSP00000479618	0.467
TBP	FKBP4	ENSP00000375942	ENSP00000001008	0.449
FKBP4	TBP	ENSP00000001008	ENSP00000375942	0.449
TBP	CEBPB	ENSP00000375942	ENSP00000305422	0.448
CEBPB	TBP	ENSP00000305422	ENSP00000375942	0.448
YY1	NR3C1	ENSP00000262238	ENSP00000231509	0.443
XBP1	RXRA	ENSP00000216037	ENSP00000419692	0.443
RXRA	XBP1	ENSP00000419692	ENSP00000216037	0.443
NR3C1	YY1	ENSP00000231509	ENSP00000262238	0.443
NR3C1	GATA3	ENSP00000231509	ENSP00000368632	0.438
GATA3	NR3C1	ENSP00000368632	ENSP00000231509	0.438
PAX5	CEBPB	ENSP00000350844	ENSP00000305422	0.416
CEBPB	PAX5	ENSP00000305422	ENSP00000350844	0.416

We firstly used siRNA specifically targeting FKBP4 in T47D and BT549 cells, and found it led to upregulation of NR3C1 at protein level (**Figures 2C, D**). In addition, we transfected FKBP4-HA plasmid in T47D and BT549 cells, which resulted in downregulation of endogenous NR3C1 (**Figures 2E, F**). These results clearly indicated that FKBP4 had a role in negatively regulating NR3C1 protein expression. Bioinformatics results also suggested a significant negative association between FKBP4 and NR3C1 both in luminal A and basal-like subtype of BC patients (**Figures S6A, B**). Furthermore, we found naringenin could upregulate NR3C1 protein expression in T47D and BT549 cells (**Figure S7**).

### NR3C1 Positively Regulates NRF2 at Protein Level

We further confirmed the specific interaction between NR3C1 and NRF2 at the molecular level. We firstly used siRNA specifically targeting NR3C1 in T47D and BT549 cells, which led to downregulation of NRF2 at protein level (**Figures 2G, H**). In addition, we transfected NR3C1-HA plasmid in T47D and BT549 cells, which resulted in upregulation of endogenous NRF2 (**Figures 2I, J**). These results clearly indicated that NR3C1 had a role in positively regulating NRF2 protein expression. Given NR3C1 was demonstrated to regulate NRF2 at protein level, we wondered whether it affected the mRNA level of NRF2. We then found silencing of NR3C1 could not lead to downregulation of NRF2 at mRNA level in T47D and BT549 cells (**Figures 2K, L**). Whereas overexpressed NR3C1 neither resulted in upregulation of NRF2 at mRNA level in T47D and BT549 cells (**Figures 2M, N**).

Meanwhile, we verified a significant positive association between NR3C1 and NRF2 both in luminal A and basal-like subtype of BC patients (**Figures S6C, D**). Downregulated NR3C1 was significantly related to four molecular subtype patients than the normal group in two databases (**Figure S8**) and was strongly correlated with worse survival both in luminal A and basal-like subtype of BC patients (**Figure S9**). Hence, our results implied that FKBP4 might downregulate NRF2 by inhibiting NR3C1 at protein level.

### FKBP4 Binds to NR3C1 and Regulates Nuclear Translocation of NR3C1

**Figure 3:**
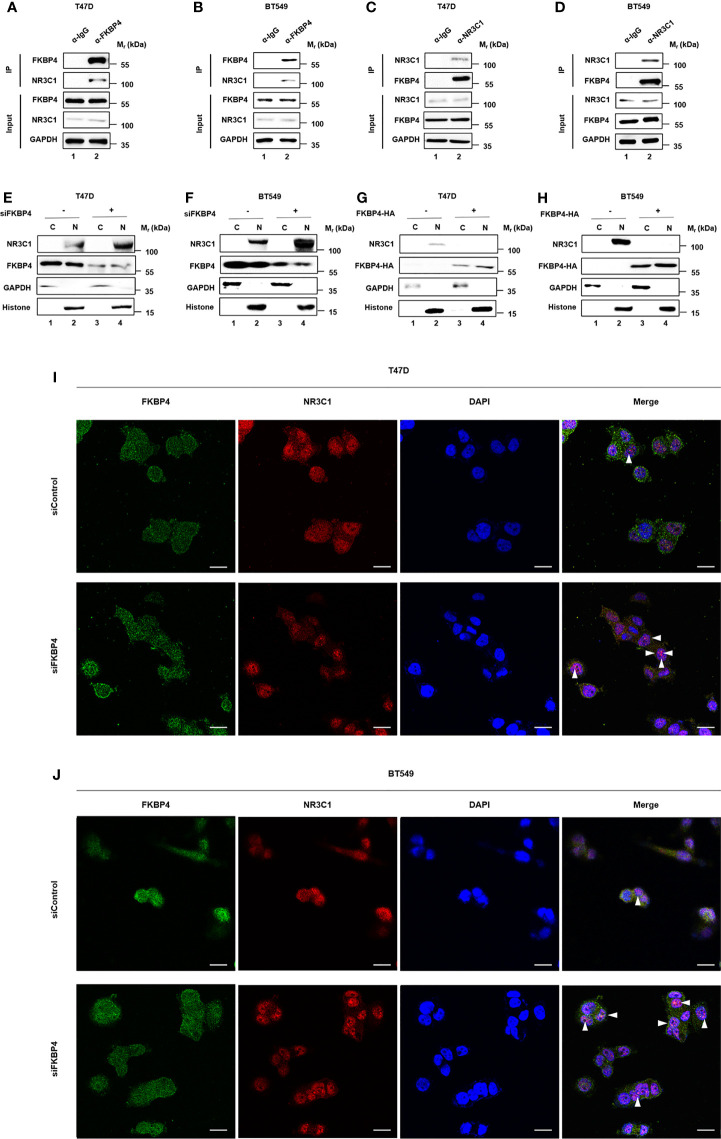
FKBP4 binds to NR3C1 and FKBP4 inhibition triggers nuclear translocation of NR3C1. **(A–D)** co-IP and western blot assays using antibodies as indicated for binding between endogenous FKBP4 and NR3C1 in T47D and BT549 cells. **(E–H)** T47D and BT549 cells were transfected with siRNAs or plasmids. Cells were harvested, and the nuclear and cytoplasmic fractions were separated. Proteins were analyzed by immunoblotting using anti-NR3C1, anti-FKBP4, anti- GAPDH, or anti-histone antibody. **(I, J)** T47D and BT549 cells were transfected with FKBP4 siRNAs. Immunofluorescent staining was carried out using anti-NR3C1 and anti-FKBP4. DAPI staining was performed to show the nuclei (n = 3 independent biological replicates). The arrowheads show the NR3C1 nuclear localization under FKBP4 inhibition. Scale bar = 20 μm.

As FKBP4 regulates its cellular targets *via* protein–protein interaction (36, 37), we tested whether FKBP4 bound to NR3C1. We performed co-IP and western blot assays using BT549 and T47D cells, and results showed that FKBP4 and NR3C1 bound with each other (**Figures 3A–D**). We further observed that nuclear accumulations of NR3C1 were enhanced by siRNA targeting FKBP4 or inhibited by FKBP4-HA plasmid in BT549 and T47D cells by western blot analysis (**Figures 3E–H**). Silencing FKBP4 mediated promotion of NR3C1 nuclear accumulations were also observed in BT549 and T47D cells by immunofluorescence microscopy (**Figures 3I, J**).

These observations were consistent with the finding that FKBP4 inhibited NRF2 through interacting with NR3C1 at protein level.

### FKBP4/NR3C1/NRF2 Signaling Pathway Involved in Naringenin-Restrained Autophagy

**Figure 4:**
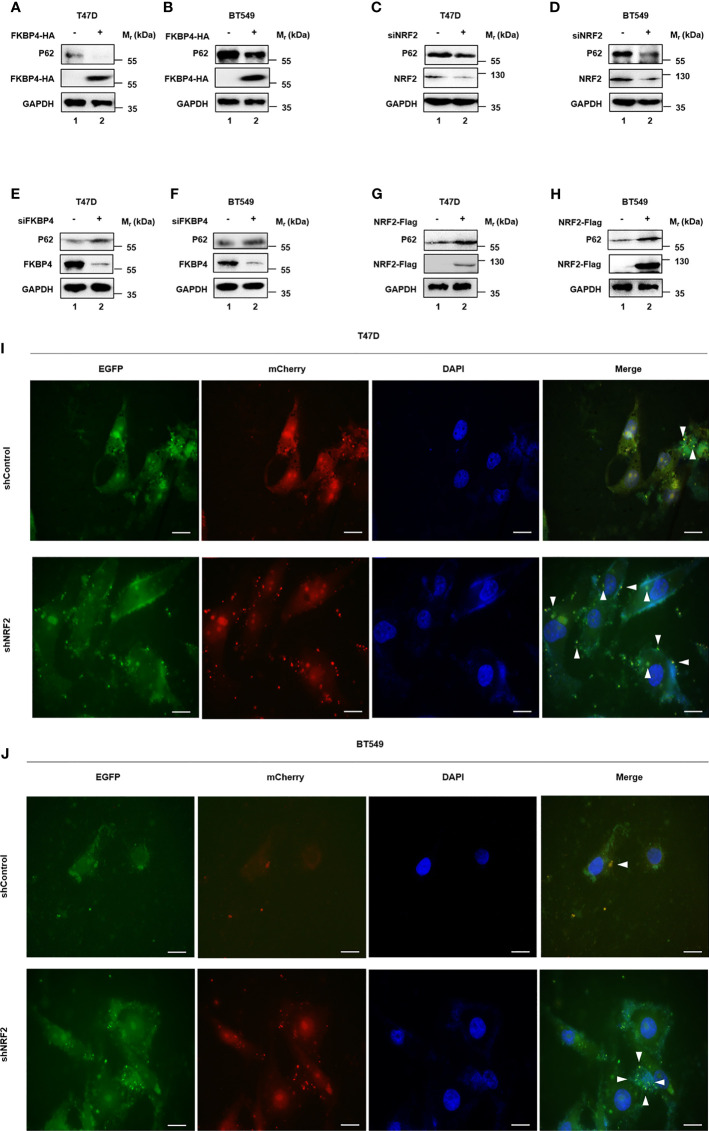
FKBP4/NR3C1/NRF2 axis is involved in naringenin-restrained autophagy. **(A, B)** Representative western blot analysis results of P62, FKBP4-HA, and endogenous control GAPDH. Western blot analysis showed overexpressing FKBP4 resulted in downregulation of P62 in T47D and BT549 cells. **(C, D)** Representative western blot analysis results of P62, NRF2, and endogenous control GAPDH. Western blot analysis showed silencing NRF2 resulted in downregulation of P62 in T47D and BT549 cells. **(E, F)** Representative western blot analysis results of P62, FKBP4, and endogenous control GAPDH. Western blot analysis showed silencing FKBP4 resulted in upregulation of P62 in T47D and BT549 cells. **(G, H)** Representative western blot analysis results of P62, NRF2-Flag, and endogenous control GAPDH. Western blot analysis showed overexpressing NRF2 resulted in upregulation of P62 in T47D and BT549 cells. **(I, J)** Measurement of autophagy flux in T47D and BT549 cells co−transfected with p−mCherry−C1−EGFP−hLC3B and shControl or shNRF2. Scale bar = 20 μm.

Since naringenin had been used to inhibit autophagy (38), we doubted whether naringenin related FKBP4/NR3C1/NRF2 axis was also involved in autophagy. Western blotting showed that FKBP4 overexpression decreased the expression of autophagy associated molecule P62 in T47D and BT549 cells (**Figures 4A, B**). Similarly, silencing NRF2 in T47D and BT549 cells led to the downregulation of P62 (**Figures 4C, D**). Meanwhile, western blotting showed that silencing FKBP4 increased the expression of P62 in T47D and BT549 cells (**Figures 4E, F**), and NRF2 overexpression led to the upregulation of P62 (**Figures 4G, H**). As shown in **Figure S10**, NR3C1 had the same effects as NRF2 on P62. Autophagy flux was also observed increased in T47D and BT549 cells silencing NRF2 (**Figures 4I, J**).

All together, these results suggested that naringenin enhanced BC cell autophagy partially owing to FKBP4/NR3C1/NRF2 axis.

### FKBP4/NR3C1/NRF2 Signaling Pathway Involved in Naringenin-Restrained Cell Proliferation

**Figure 5:**
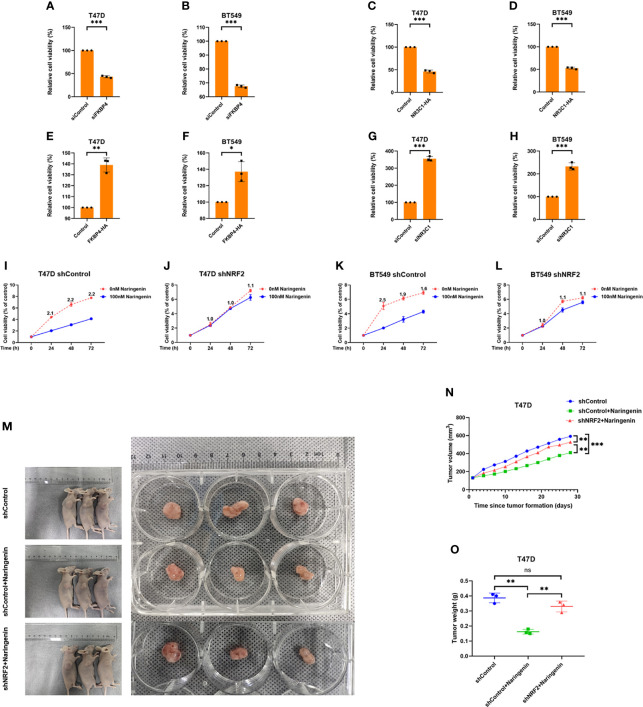
FKBP4/NR3C1/NRF2 axis is involved in naringenin-restrained cell proliferation. **(A, B)** Bar chart showed silencing FKBP4 resulted in decreased cell viability of T47D and BT549 cells at 72 h (n = 3 independent biological replicates). **(C, D)** Bar chart showed overexpressing NR3C1 resulted in decreased cell viability of T47D and BT549 cells at 72 h (n = 3 independent biological replicates). **(E, F)** Bar chart showed overexpressing FKBP4 resulted in increased cell viability of T47D and BT549 cells at 72 h (n = 3 independent biological replicates). **(G, H)** Bar chart showed silencing NR3C1 resulted in increased cell viability of T47D and BT549 cells at 72 h (n = 3 independent biological replicates). **(I–L)** Line chart showed knockdown of NRF2 by shRNA attenuated naringenin-restrained cell proliferation in T47D and BT549 cells treated by 100 nM naringenin, numeric representation at each dot is cell viability fold change of 0 and 100 nM naringenin treatment (n = 3 independent biological replicates). **(M–O)** The macroscopic appearance, volume and weight of subcutaneous tumors in mice (n = 3/group) transplanted with T47D cells treated with shControl, shControl + Naringenin, and shNRF2 + Naringenin. *p <0.05, **p <0.01, ***p <0.001, ns, nonsense.

While naringenin is well-known to inhibit cell proliferation (39), we doubted whether naringenin related FKBP4/NR3C1/NRF2 axis was also involved in cell proliferation. Cell viability assay showed that silencing FKBP4 or overexpressing NR3C1 prevented cell proliferation of both T47D and BT549 cells at 72 h (**Figures 5A–D**), while overexpressing FKBP4 or silencing NR3C1 promoted cell proliferation of both T47D and BT549 cells at 72 h (**Figures 5E–H**). Additionally, knockdown of NRF2 significantly prevented naringenin-restrained cell proliferation of T47D and BT549 cells during 72 h (**Figures 5I–L**). These findings were further confirmed by cell photography (**Figure S11**) and colony formation assay (**Figure S12**).

T47D cells treated with silenced NRF2 were used to generate subcutaneous xenograft models in nude mice. Results showed that the volume and weight of tumors were significantly reduced in the naringenin treated group than in the control group, whereas those in the naringenin + shNRF2 group were significantly greater than in the naringenin treated group (**Figures 5M–O**).

Thus, *in vivo and in vitro* experiments indicated that naringenin inhibited BC proliferation partially owing to the FKBP4/NR3C1/NRF2 signaling pathway.

### FKBP4/NR3C1/NRF2 Signaling Pathway Involved in Naringenin-Induced DC Differentiation and Maturation

**Figure 6:**
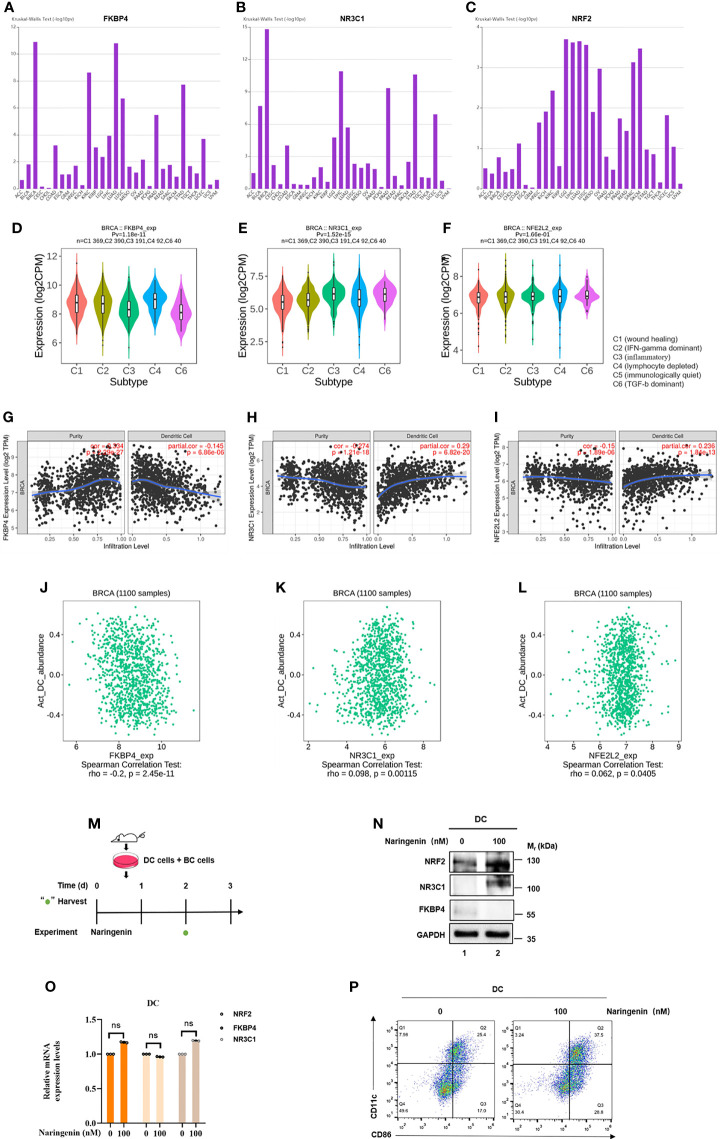
FKBP4/NR3C1/NRF2 signaling pathway involved in naringenin-induced DC differentiation and maturation. **(A–C)** Distribution of FKBP4, NR3C1, and NRF2 expression across immune subtypes in pan-cancer analysis using TISIDB database. **(D–F)** Distribution of FKBP4, NR3C1, and NRF2 expression across immune subtypes only in breast cancer using TISIDB database. **(G–I)** Spearman correlations between expression of FKBP4, NR3C1 or NRF2 and infiltration level of DCs in breast cancer using TIMER database. **(J–L)** Spearman correlations between expression of FKBP4, NR3C1 or NRF2 and abundance of DCs in breast cancer using TISIDB database. **(M)** Schema of mice DC cells co-cultured with BC cells treated by naringenin. **(N)** Western blot analysis of NR3C1, FKBP4, and NRF2 protein expression in DCs co-cultured with BC cells for 3 days. **(O)** RT-qPCR analysis of NR3C1, FKBP4, and NRF2 mRNA expression in DCs co-cultured with BC cells for 3 days. **(P)** DC cells were treated with naringenin for 72 h and analyzed for maturation by flow cytometry analysis. ns, nonsense.

Since naringenin had been found connected with the immune system (40), we investigated the correlations between FKBP4/NR3C1/NRF2 signaling pathway and various immune signatures using TISIDB database and TIMER database. **Figures 6A–F** showed that associations between FKBP4, NR3C1, and NRF2 expression and immune subtypes across human cancers, FKBP4 and NR3C1 were most closely associated with immune subtypes of BC. Similar to the finding of Liang et al. that naringenin induced more DCs infiltration into tumor (41), we firstly mined the relationship of FKBP4/NR3C1/NRF2 axis and DC infiltration level and abundance, namely, FKBP4 expression was significantly negatively correlated with DC infiltration level and abundance, while NR3C1 and NRF2 expression were significantly positively correlated with DC infiltration level and abundance in two databases (**Figures 6G–L**). However, FKBP4/NR3C1/NRF2 signaling pathway did not affect the relationship between DC infiltration and prognosis of BC, nor did their mutations in DC cells affect DC infiltration level in BC (**Figure S13**).

Next, to confirm if FKBP4/NR3C1/NRF2 axis was truly regulated in DC, we co-cultured BC cells and bone marrow cells of mice with GMCSF + IL4, simulated as DC (**Figure 6M**). After naringenin treatment for 72 h, we found that FKBP4 was decreased while NR3C1 and NRF2 were increased at protein but not mRNA level (**Figures 6N, O**). Flow cytometry analysis results showed that the positive expression percentages of CD11c and CD86 of DC were significantly increased in the naringenin group in contrast to those in the control group (**Figure 6P**). These data suggested that FKBP4/NR3C1/NRF2 signaling pathway was partially involved in naringenin-induced DC differentiation and maturation.

**Figure 7:**
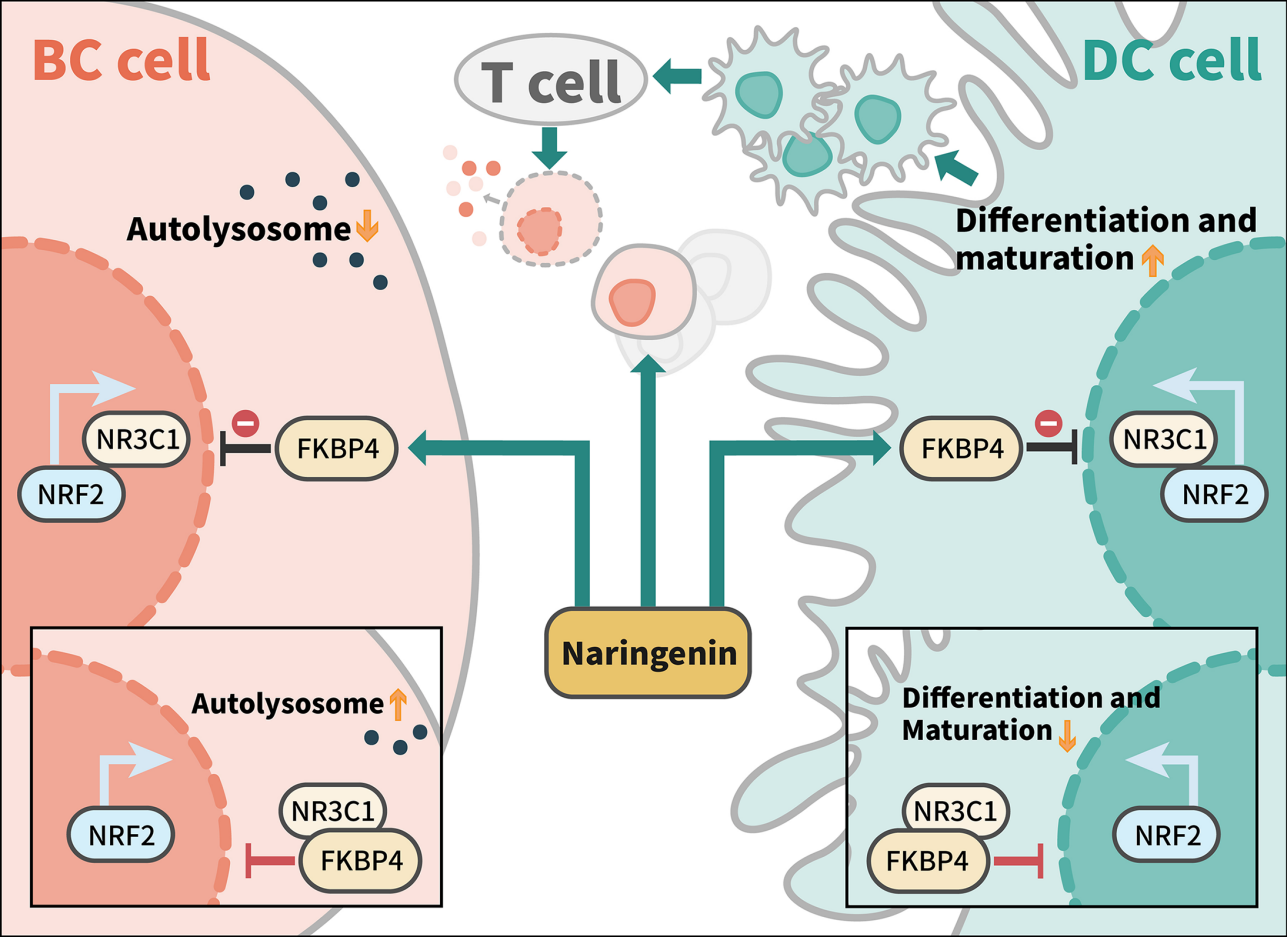
Model of naringenin-mediated inhibition of autophagy and cell proliferation and promotion of DC differentiation and maturation through FKBP4/NR3C1/NRF2 signaling pathway. Naringenin inhibits FKBP4 protein, thus releasing and inducing NR3C1 protein to bind to NRF2 protein, leading to autophagy and proliferation inhibition of BC cells, as well as differentiation and maturation promotion of DC. Among tumor microenvironment, naringenin might promote T cells to kill breast cancer cells by enhancing the antigen presentation function of DC cells.

Taken together, we demonstrated that naringenin mediated anti-autophagy, anti-proliferation of BC cells, and pro-DC differentiation and maturation in a new-found FKBP4/NR3C1/NRF2 dependent way (**Figure 7**).

## Discussion

Endogenous host-derived regulators of NRF2 have been becoming the hotspot on related researches. For instance, when specific thiol residues are modified by electrophiles, NRF2 is released from the E3 ligase adaptor Kelch-like ECH-associated protein 1 (Keap1) related complex and translocates into the nucleus wherein it performs its functions (42). Yu et al. defined the Bax-binding protein MOAP-1 as a negative regulator of NRF2 *via* dissociation of p62 bodies (43). TRIM25 expression was found positively associated with NRF2 expression in hepatocellular carcinoma, mechanistically, TRIM25 directly targeted Keap1 by ubiquitination and degradation, leading to NRF2 activation (44). The current study firstly suggested that FKBP4/NR3C1 axis might be a novel endogenous negative regulator of NRF2 by altering its post-transcriptional activity in both BC cells and DCs. Thus, FKBP4 and NR3C1 could serve as notable therapeutic molecules against NRF2-dependent tumorigenesis, autoinflammation, and autoimmunity in the future.

FK506-binding protein family in human genomes has included 18 FKBPs up to date, which could target on various pathways in embryonic development, stress response, cardiac function, cancer tumorigenesis, and neuronal function (45). In colorectal cancer, silencing FKBP3 has been found to attenuate oxaliplatin resistance by regulation of the phosphatase and tensin homolog (PTEN)/AKT axis (46). In Alzheimer’s disease, FKBP12 and amyloid precursor protein (APP) interplay has been suspected to affect Aβ peptides expression (47). Although FKBP4 has been demonstrated to connect mammalian target of naringenin complex 2 (mTORC2) and phosphoinositide-3-kinase (PI3K) to enhance cell proliferation of BC, for the first time, we found that FKBP4 played a carcinogenic role by downregulating NRF2 in BC cells and co-cultured DCs.

Currently, there is no research of regulation mechanisms on NR3C1 and NRF2, but only a few studies suggest that NR3C1 is involved in the innate immune response (48, 49). In our study, we demonstrated FKBP4 post-transcriptionally downregulated NRF2 through binding to NR3C1, thus inhibiting nuclear translocation of NR3C1, but this effect was found relatively weak because shNR3C1 could not completely inhibit FKBP4 from regulating NRF2 (data not shown). Therefore, although we speculated that some of the predicted TFs were also involved in FKBP4/NRF2 pathway, subsequent mechanism studies need to be further improved to verify our conjecture.

As for the interaction of FKBP and autophagy, FKBP5 has been reported to change phosphorylation of Beclin1 by binding to Beclin1, thus triggering autophagic pathways (50). FKBP8 was found to recruit lipidated LC3A to induce mitochondrial autophagy *via* N-terminal LC3-interacting region motif of FKBP8 (51), while the regulation of autophagy by FKBP4 in tumor cells has not been reported yet. Meanwhile, NRF2 was also involved in autophagy, e.g., Moscat et al. demonstrated that protein kinase C (PKC) λ/ι loss in hepatocytes could promote autophagy and oxidative phosphorylation, through which NRF2 drove cancer cell through cell-autonomous and non-autonomous mechanisms (52). In the current study, we first demonstrated that inhibiting FKBP4 could weaken autophagy by increasing NRF2 expression of BC cells, thus autophagy plays an oncogenic role in this context. Actually, autophagy plays a dual role depending on the different types of context of BC (53). For instance, inhibition of autophagy along with HER2 inhibition is critical for promoting BC regression, but autophagy stimulation could transform the effectiveness of HER2 treatments (54), Meanwhile small-molecule RL71 triggered excessive autophagic cell death as a potential therapeutic strategy in triple-negative BC (55), implying that autophagy could function as a tumor suppressive mechanism. Additionally, autophagy seems to exert a dual role in luminal breast cancer, and also triple-negative BC (56).

Takabe et al. demonstrated that high NRF2 tumors were associated with high infiltration of DCs by conducting *in silico* analyses in 5,443 breast cancer patients from several large patient cohorts (57). Besides, it is likely that different methods mediate the transfer of cargos to DCs through NRF2 related signalings, e.g., the project that our team is currently working on has found that extracellular vesicles secreted by naringenin treated BC cells induced DC differentiation and maturation on NRF2 dependent way (data not shown). Further experimentation will be required to determine the relative contribution of these different mechanisms of tumor DNA delivery to DCs.

Recently, a growing body of evidence has demonstrated that the pharmacological agents, e.g., Ezetimibe ketone, Desfluoro-ezetimibe, NK-252, Bardoxolone, and TBHQ could induce activation of NRF2 (58–61). Here we proposed naringenin, a citrus flavonoid shown to have cytotoxic and antiproliferative effects on various cancer cell types (62) and regulates immunological pathways (63), to be another kind of potential NRF2 conditioning agent. With the development of immunotherapies such as cancer vaccine, immune checkpoint inhibitors, oncolytic virus, and chimeric antigen receptor T cell (CAR-T) therapy (64), a combination of NRF2-targeting agonists and immunotherapies may provide multiple feasible approaches to new BC treatment strategies.

## Data Availability Statement

The raw data supporting the conclusions of this article will be made available by the authors, without undue reservation.

## Ethics Statement

The animal study was reviewed and approved by the Animal Welfare Ethics Committee of Zhejiang Provincial People’s Hospital.

## Author Contributions

HX designed the experiment. HX and CZ performed most of experiments. BL, BX, XL, CC, and ZL performed the western blot assay and the real-time PCR experiments. YuJ, ZW, MY, and YoJ contributed to bioinformatics analysis. HX, ZC, and BL analyzed the data and wrote the paper. HX, LW, JZ and XM conducted the study supervision. All authors contributed to the article and approved the submitted version.

## Funding

The work was supported by the National Natural Science Foundation of China (No. 81972453, No. 81972597, No. 82000212 and No. 82102814), Zhejiang Provincial Natural Science Foundation of China under grants (No. LY19H160055, LY19H160059, LY20H160026, LQ21H160022 and LQ22H160053). The work was sponsored by Zhejiang Provincial Medical and Health Science and Technology Project (No. 2018ZD028 and No. 2021RC003), Zhejiang Provincial People’s Hospital Scientific Research Foundation for The Excellent Youth (ZRY2020B007).

## Conflict of Interest

The authors declare that the research was conducted in the absence of any commercial or financial relationships that could be construed as a potential conflict of interest.

## Publisher’s Note

All claims expressed in this article are solely those of the authors and do not necessarily represent those of their affiliated organizations, or those of the publisher, the editors and the reviewers. Any product that may be evaluated in this article, or claim that may be made by its manufacturer, is not guaranteed or endorsed by the publisher.
