# Supplementary material for: Naringenin Regulates FKBP4/NR3C1/NRF2 Axis in Autophagy and Proliferation of Breast Cancer and Differentiation and Maturation of Dendritic Cell
Source: Front Immunol. 2022 Jan 11;12:745111. doi: 10.3389/fimmu.2021.745111 (PMC8786807; doi:10.3389/fimmu.2021.745111)
Supplement: Supplementary file 1 [file DataSheet_1.pdf]

Supplementary figures

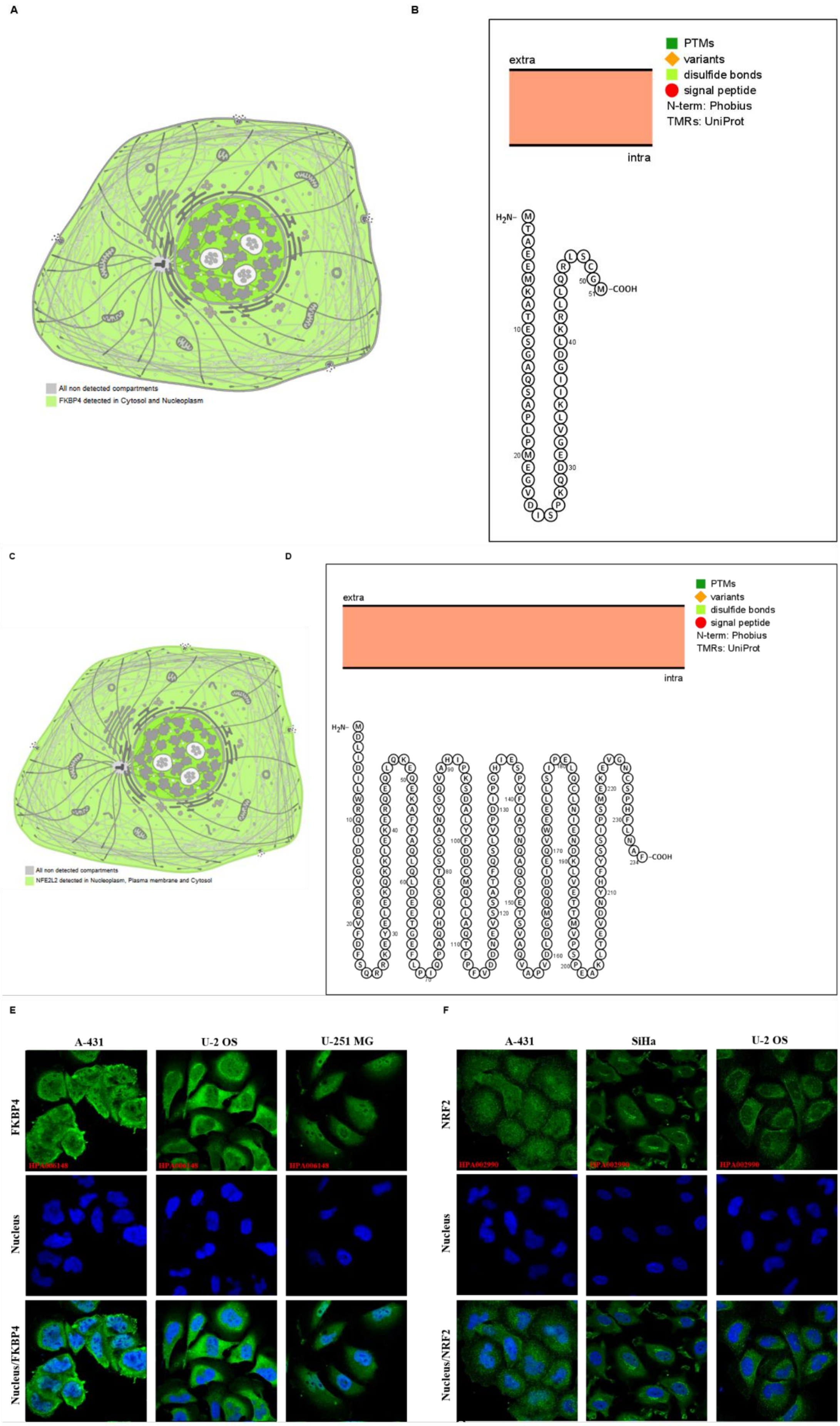
A
B
extra
intra
PTMs
variants
disulfide bonds
signal peptide
N-term: Phobius
TMRs: UniProt
All non detected compartments
FKBP4 detected in Cytosol and Nucleoplasm
C
D
NFE2L2 detected in Nucleoplasm, Plasma membrane and Cytosol
E
F
A-431
U-2 OS
U-251 MG
SiHa
FKBP4
NRF2
Nucleus
Nucleus/FKBP4
Nucleus/NRF2

Figure 2

Figure S1 Localization of FKBP4 and NRF2. **a**, **b** FKBP4 protein localization and topology showing cytosol and nucleoplasm localization. **c**, **d** NRF2 protein localization and topology showing nucleoplasm, plasma membrane and cytosol localization. **e** Immunofluorescence staining of the subcellular distribution of FKBP4 within the nucleus of A-431, U-2 OS and U-251 MG cells as adopted from the HPA database. **f** Immunofluorescence staining of the subcellular distribution of NRF2 within the nucleus of A-431, SiHa and U-2 OS cells as adopted from the HPA database.

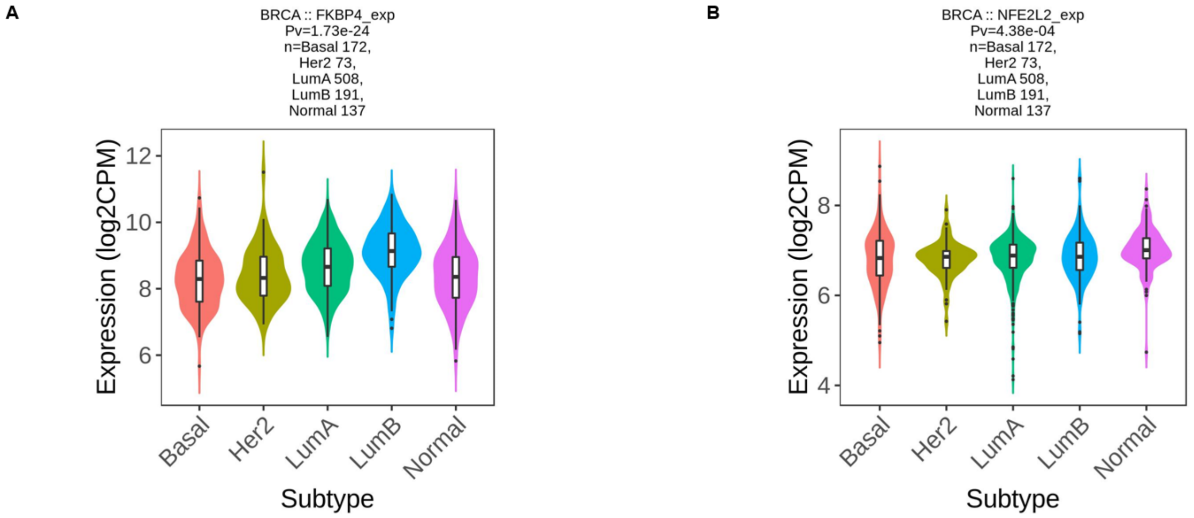

Figure S2 Expression of FKBP4 and NRF2 in four subtypes of BC patients using TISIDB database. **a** A violin plot indicated upregulated FKBP4 in luminal A, luminal B, HER2-positive and basal-like subtype of BC patients than the normal group. **b** A violin plot indicated downregulated NRF2 in luminal A, luminal B, HER2-positive and basal-like subtype of BC patients than the normal group.

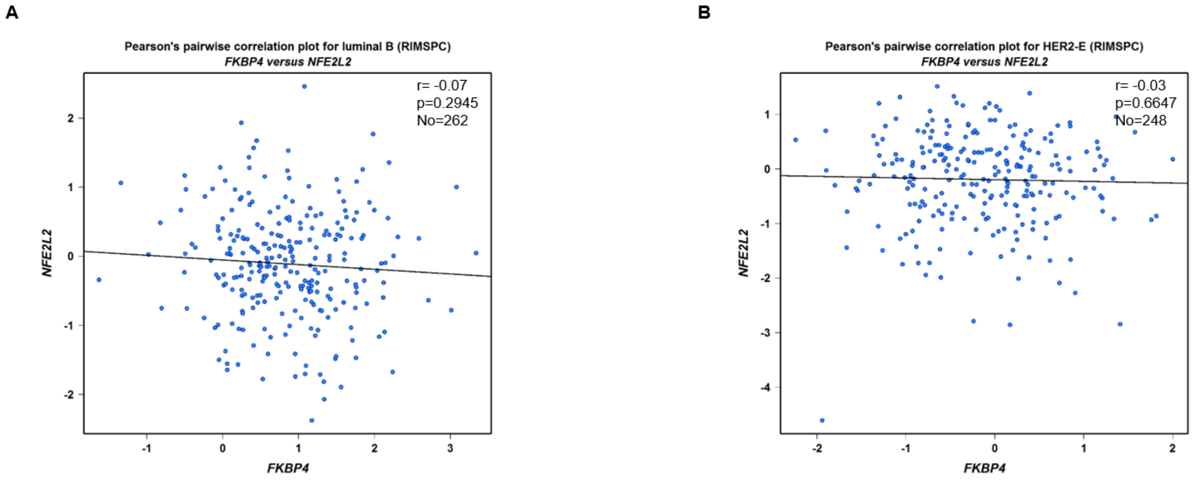

Figure S3 Pearson's pairwise correlation plot of FKBP4 and NRF2 in luminal B and HER2-positive subtype of BC patients. **a** Pearson's pairwise correlation plot of FKBP4 and NRF2 in luminal B subtype of BC patients, r= -0.07, p=0.2945, No=262. **b** Pearson's pairwise correlation plot of FKBP4 and NRF2 in HER2-positive subtype of BC patients, r= -0.03, p=0.6647, No=248.

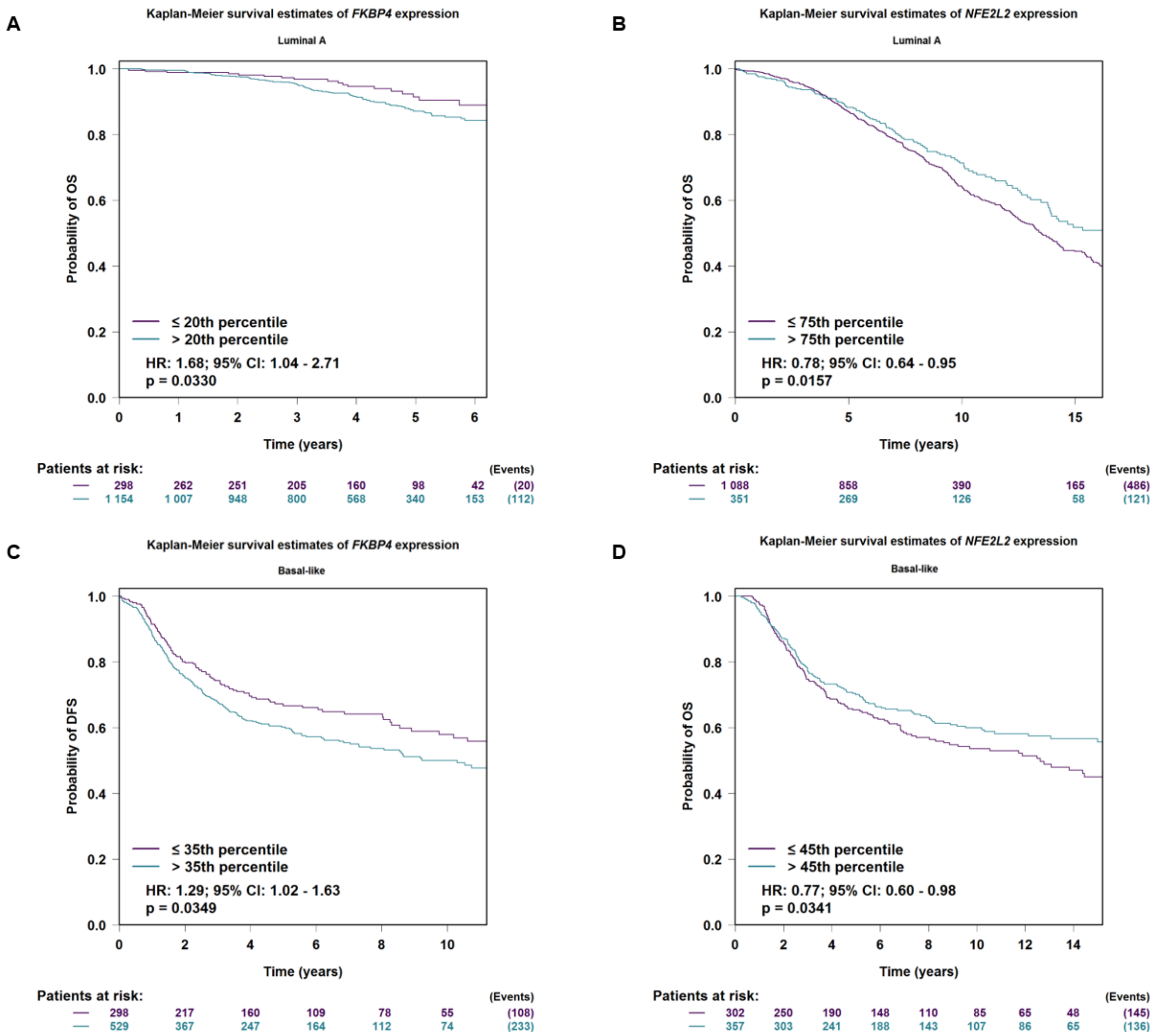

Figure S4 Kaplan-Meier survival curves of FKBP4 and NRF2 in luminal A and basal-like subtype of BC patients. **a, b** Kaplan-Meier survival curves of FKBP4 and NRF2 were plotted for luminal A subtype of BC patients, respectively. **c, d** Kaplan-Meier survival curves of FKBP4 and NRF2 were plotted for basal-like subtype of BC patients, respectively.

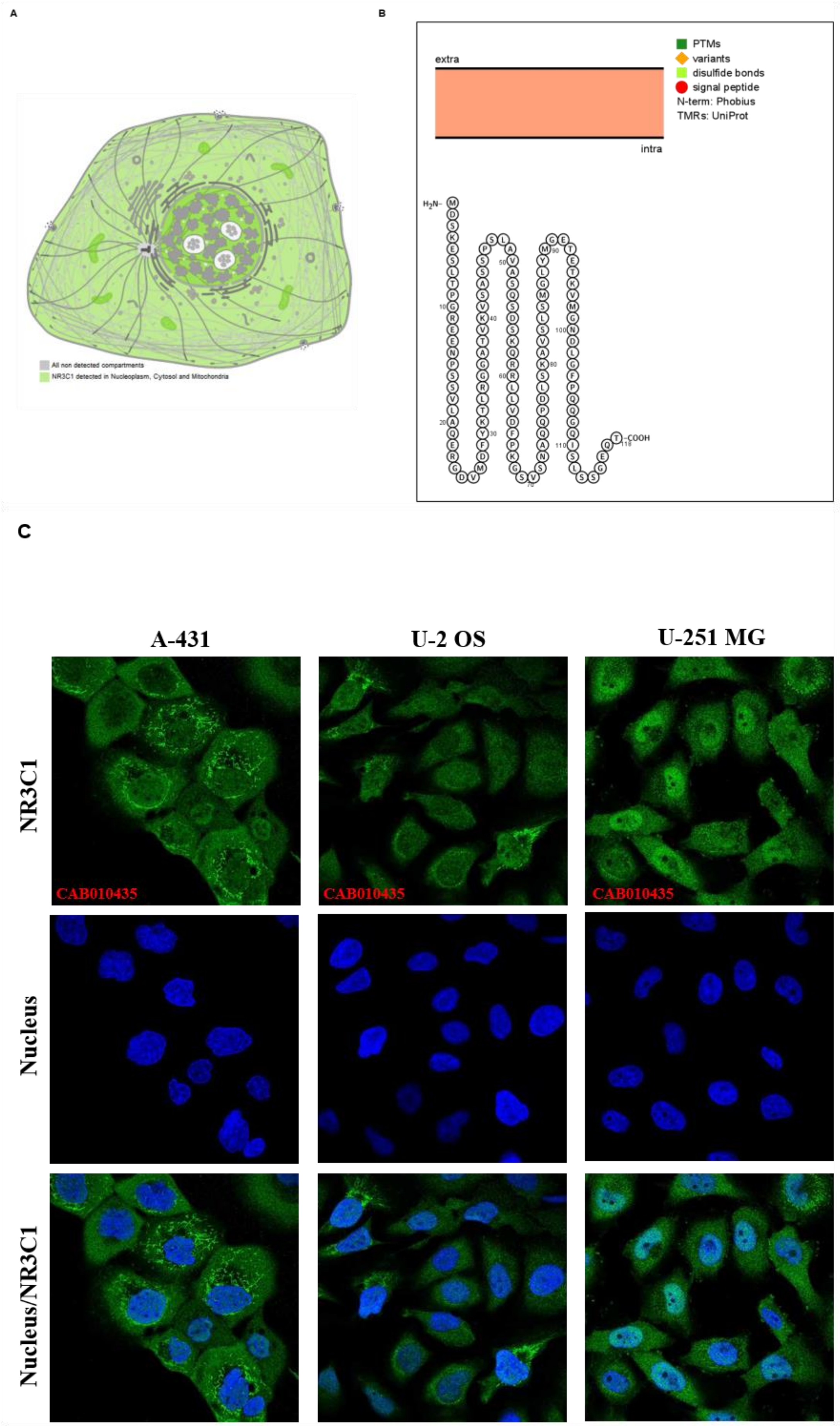
A
All non detected compartments
NR3C1 detected in Nucleoplasm, Cytosol and Mitochondria
B
extra
intra
PTMs
variants
disulfide bonds
signal peptide
N-term: Phobius
TMRs: UniProt
H2N-
-COOH
C
A-431
U-2 OS
U-251 MG
NR3C1
Nucleus
Nucleus/NR3C1
CAB010435
CAB010435
CAB010435

Figure S5 Localization of NR3C1. **a**, **b** NR3C1 protein localization and topology showing nucleoplasm, cytosol and mitochondria localization. **c** Immunofluorescence staining of the subcellular distribution of NR3C1 within the nucleus of A-431, U-2 OS and U-251 MG cells as adopted from the HPA database.

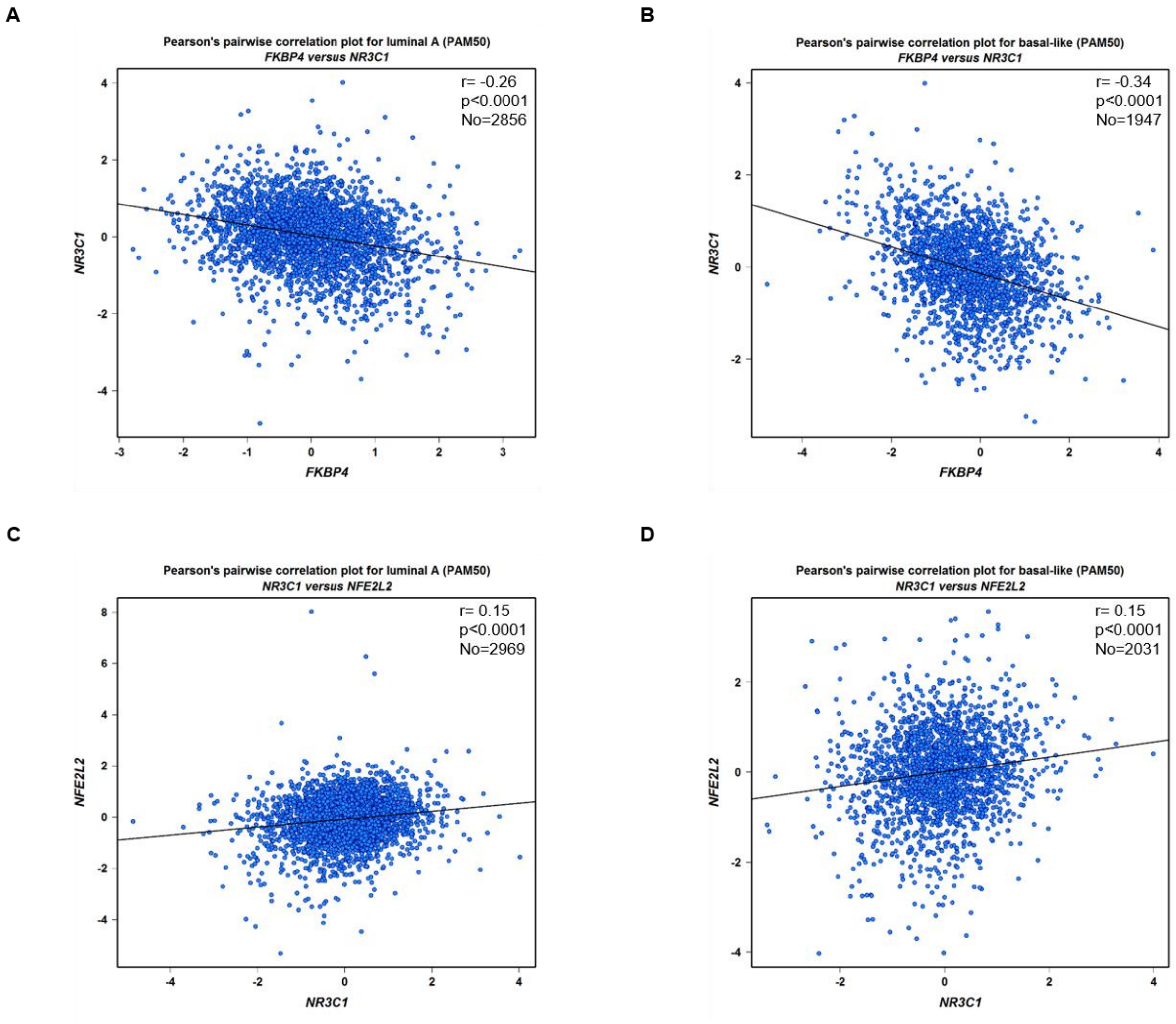

Figure S6 Pearson's pairwise correlation plot of FKBP4, NR3C1 and NRF2 in luminal A and basal-like subtype of BC patients. **a** Pearson's pairwise correlation plot of FKBP4 and NR3C1 in luminal A subtype of BC patients, r= -0.26, p<0.0001, No=2856. **b** Pearson's pairwise correlation plot of FKBP4 and NR3C1 in basal-like subtype of BC patients, r= -0.34, p<0.0001, No=1947. **c** Pearson's pairwise correlation plot of NR3C1 and NRF2 in luminal A subtype of BC patients, r=0.15, p<0.0001, No=2969. **d** Pearson's pairwise correlation plot of NR3C1 and NRF2 in basal-like subtype of BC patients, r= 0.15, p<0.0001, No=2031.

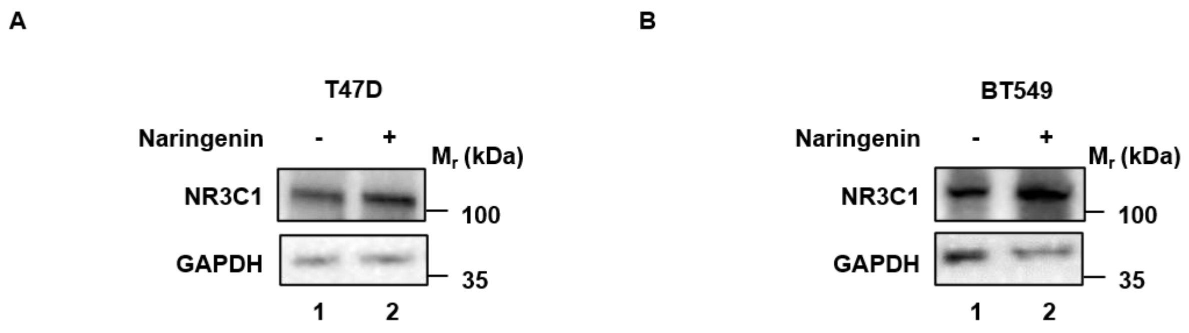

Figure S7 Naringenin upregulates NR3C1 expression in BC cells. Representative western blot analysis results of NR3C1 and endogenous control GAPDH. Western blot analysis showed upregulated NR3C1 in T47D and BT549 cells treated by 100 nM naringenin for 48 h.

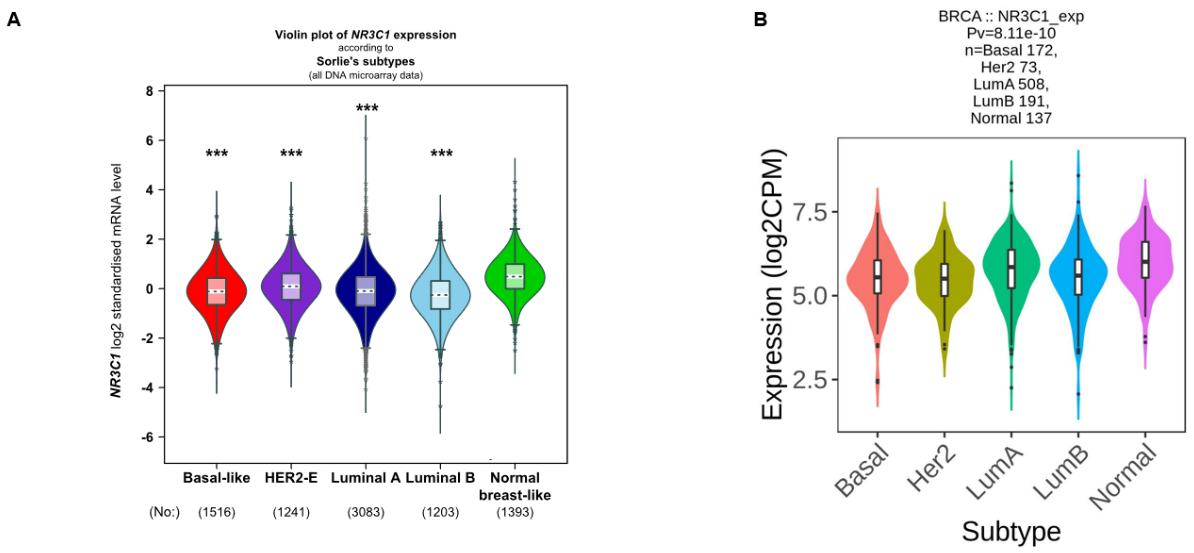

Figure S8 A violin plot of NR3C1 expression in four subtypes of BC patients and the normal group by bioinformatics analysis. ***p<0.001.

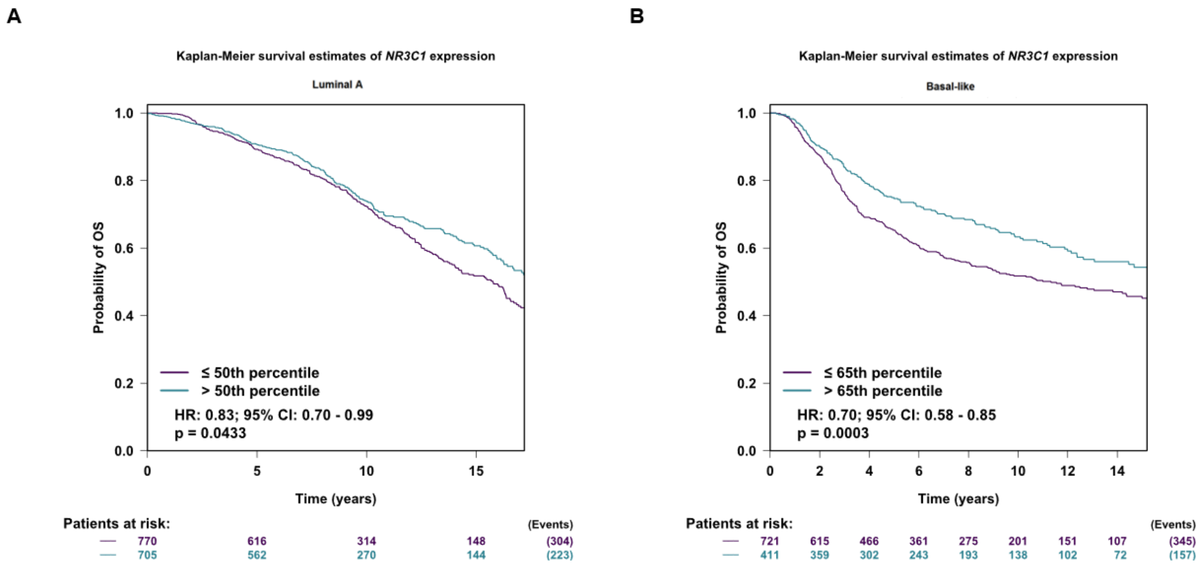

Figure S9 Kaplan-Meier survival curves of NR3C1 in luminal A and basal-like subtype of BC patients by bioinformatics analysis.

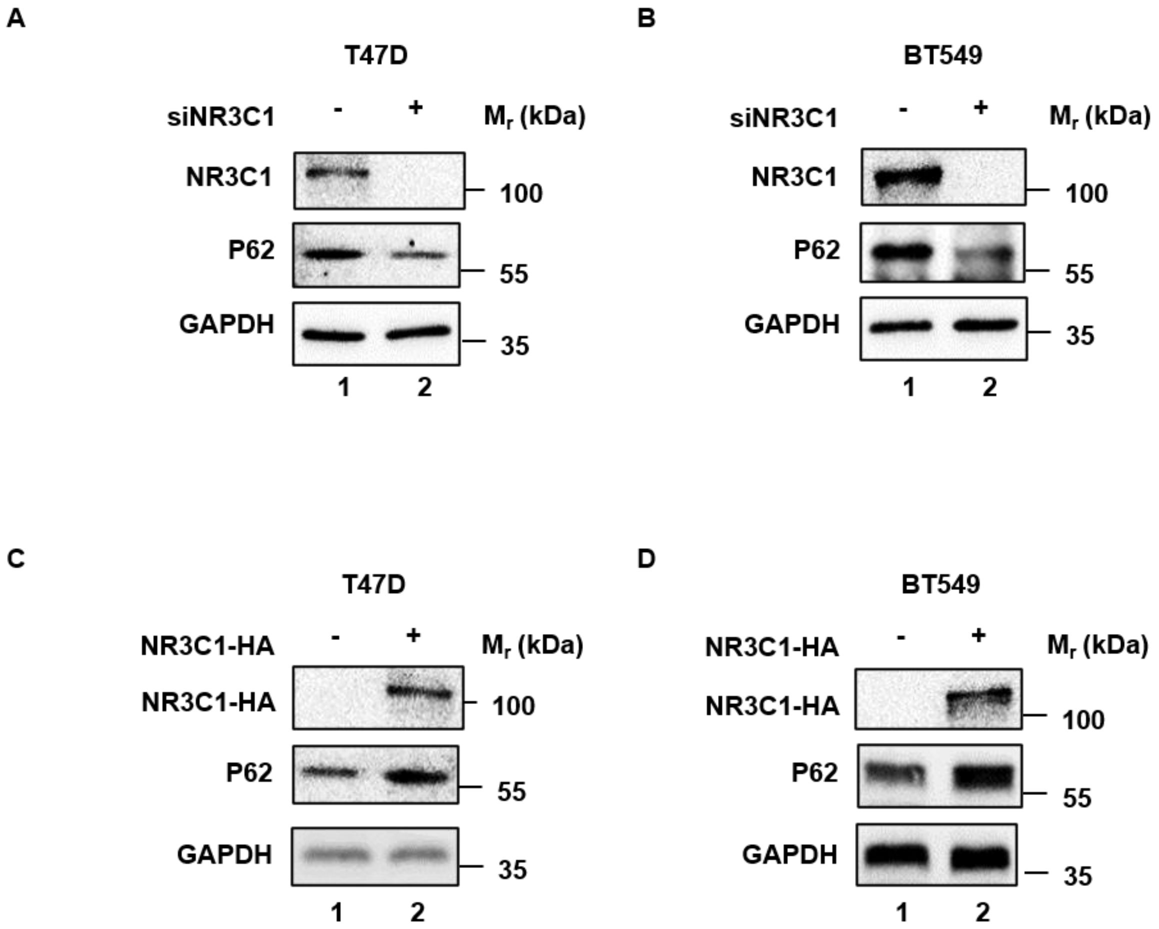

Figure S10 NR3C1 is involved in naringenin-restrained autophagy. **a, b** Representative western blot analysis results of P62, NR3C1 and endogenous control GAPDH. Western blot analysis showed silencing NR3C1 resulted in downregulation of P62 in T47D and BT549 cells. **c, d** Representative western blot analysis results of P62, NR3C1-HA and endogenous control GAPDH. Western blot analysis showed overexpressing NR3C1 resulted in upregulation of P62 in T47D and BT549 cells.

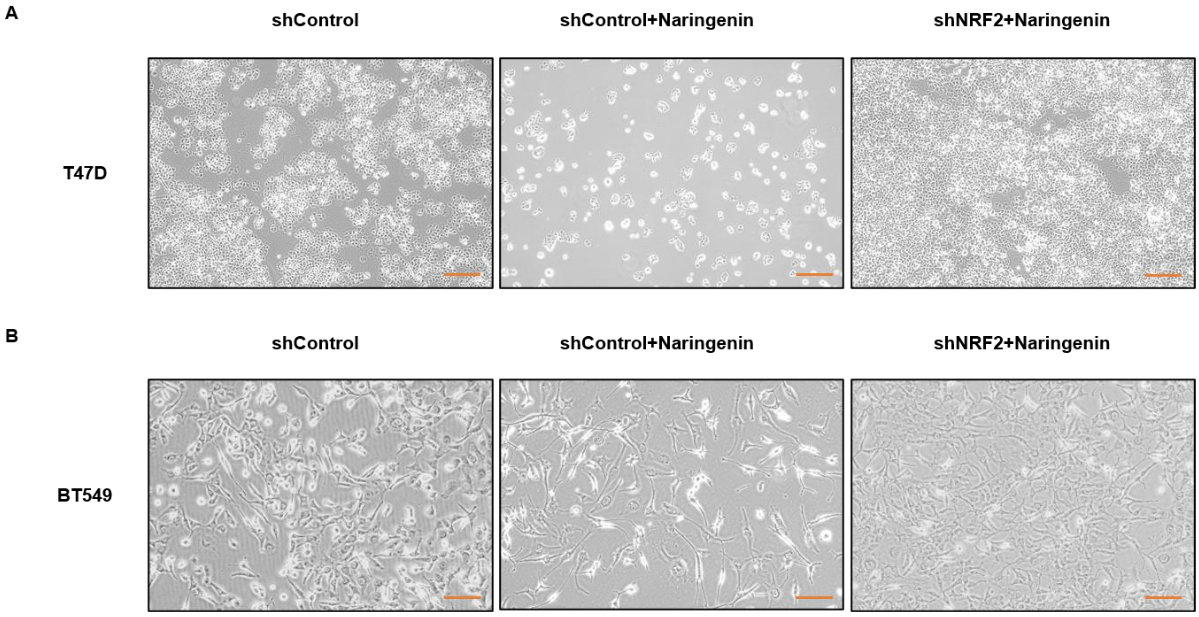

Figure S11 Knockdown of NRF2 by shRNA attenuates naringenin-restrained cell numbers of BC cells. **a** Representative microscopic photos showed knockdown of NRF2 by shRNA attenuated naringenin-restrained cell numbers of T47D cells treated by 100 nM naringenin for 72 h. **b** Representative microscopic photos showed knockdown of NRF2 by shRNA attenuated naringenin-restrained cell numbers of BT549 cells treated by 100 nM naringenin for 72 h. Scale bar=50μm.

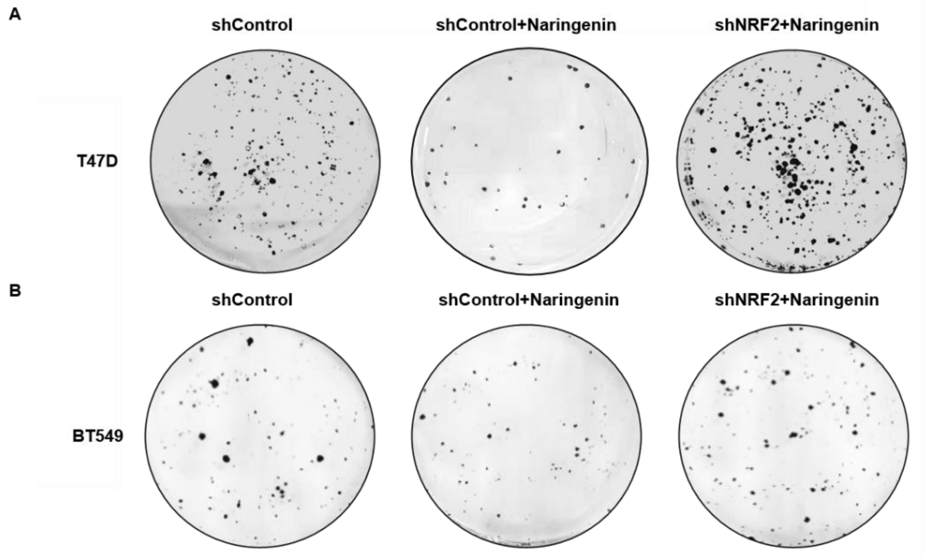

Figure S12 Knockdown of NRF2 by shRNA attenuates naringenin-restrained colony formation of BC cells. **a** Colony formation assay showed knockdown of NRF2 by shRNA attenuated naringenin-restrained colony formation of T47D cells treated by 100 nM naringenin for two weeks. **b** Colony formation assay showed knockdown of NRF2 by shRNA attenuated naringenin-restrained colony formation of BT549 cells treated by 100 nM naringenin for two weeks.

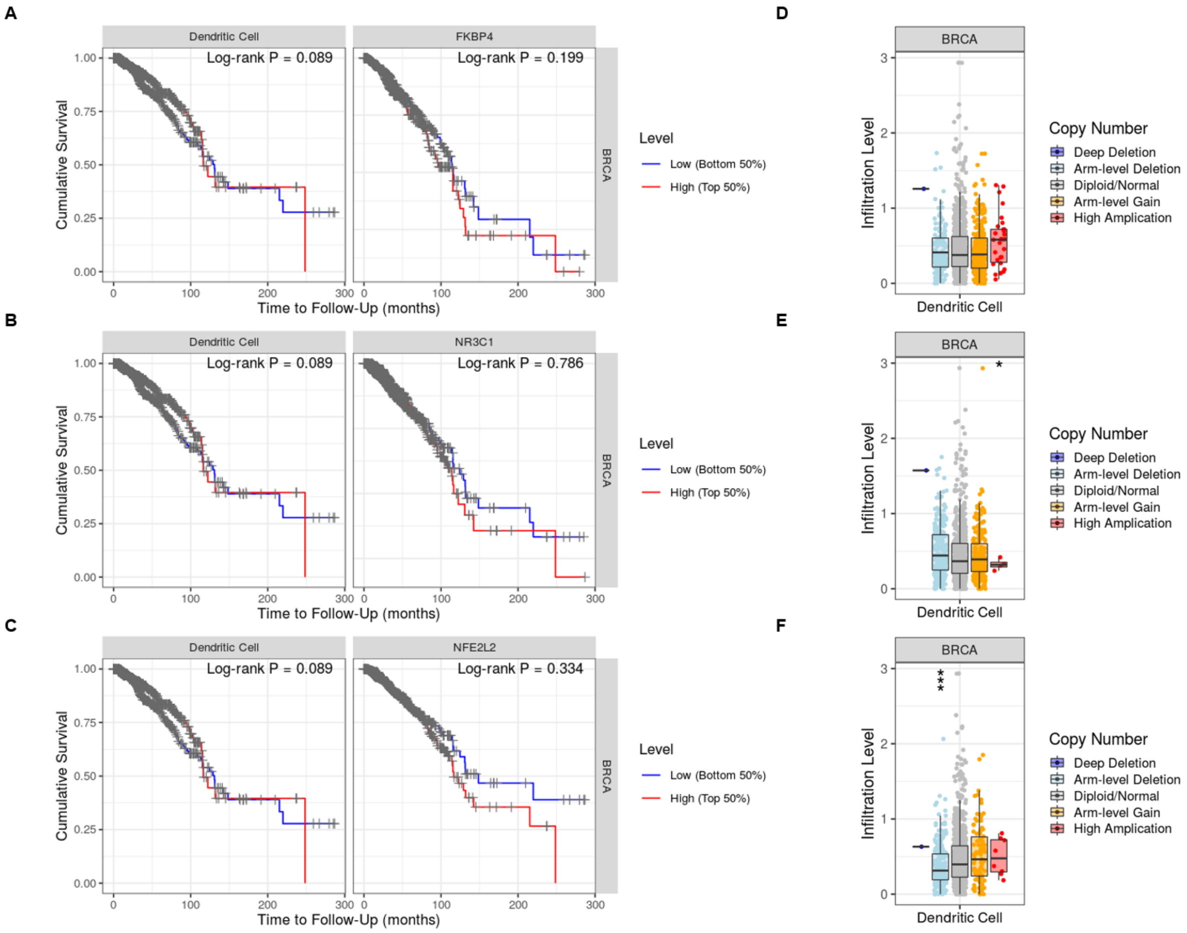

Figure S13 Relation between DC and immunological merit of FKBP4, NR3C1 or NRF2 in BC patients using TIMER database. **a-c** Kaplan-Meier plots for DC infiltrates and FKBP4, NR3C1 or NRF2 to visualize the survival differences. **d-f** Comparison of DC infiltration levels among BC with different somatic copy number alterations for FKBP4, NR3C1 or NRF2.
